# Chemotherapy enhances HMGA1 secretion through the mutant p53-CK2 axis in pancreatic ductal adenocarcinoma cells

**DOI:** 10.1038/s41419-025-08082-1

**Published:** 2025-10-27

**Authors:** Federica Danzi, Giovanna Butera, Damien Sutton, Matthew D. Perricone, Yushu Hu, Adriana Celesia, Marcello Manfredi, Jessica Brandi, Narges Pourmandi, Noah S. Nelson, Lin Lin, Michele Bevere, Raffaella Pacchiana, Antonio Pea, Roberto Salvia, Aldo Scarpa, Claudio Luchini, Daniela Cecconi, Stefano Ugel, Costas A. Lyssiotis, Alessandra Fiore, Massimo Donadelli

**Affiliations:** 1 Department of Neurosciences, Biomedicine and Movement Sciences, Section of Biochemistry, University of Verona, Verona, Italy; 2 Department of Molecular and Integrative Physiology, University of Michigan, Ann Arbor, MI USA; 3 Department of Medicine, Section of Immunology, University of Verona Hospital Trust, Verona, Italy; 4 Biological Mass Spectrometry Lab, Department of Translational Medicine, University of Piemonte Orientale, Novara, Italy; 5 Institute for Molecular and Translational Cardiology (IMTC), IRCCS Policlinico San Donato, Milan, Italy; 6 Department of Biotechnology, University of Verona, Verona, Italy; 7 ARC-Net Applied Research on Cancer Centre, University of Verona, Verona, Italy; 8 Unit of Pancreatic Surgery, University of Verona Hospital Trust, Verona, Italy; 9 Department of Diagnostics and Public Health, Section of Pathology, University and Hospital Trust of Verona, Verona, Italy

**Keywords:** Oncogenes, Cancer

## Abstract

Pancreatic ductal adenocarcinoma (PDAC) remains one of the most aggressive and lethal cancers, with limited therapeutic options and a dismal prognosis. A critical driver of its progression is mutant p53 (mutp53), which alters the tumor microenvironment (TME) by influencing crucial pro-tumoral signaling factors. Given the potential of secretome profiling to reveal novel biomarkers and druggable targets, we investigated the role of the mutp53-driven secretome in PDAC cells and its implications for disease progression. Through mass-spectrometry (MS) analysis, we identified a set of secreted proteins modulated by mutp53, with the nuclear high mobility group A1 (HMGA1) serving as a central regulator. HMGA1 is a transcription factor involved in several cellular processes and found to be upregulated in different tumors, but its extracellular role in cancer remains largely unexplored. We demonstrate that mutp53-driven HMGA1 secretion promotes PDAC cell hyperproliferation, where HMGA1 deficiency significantly impairs tumor growth highlighting a critical role of this protein in tumor aggressiveness. Notably, we discovered that chemotherapy enhances HMGA1 secretion specifically in *TP53*-mutant PDAC cells through a mechanism dependent on Casein Kinase 2 (CK2) activity. To unravel the downstream oncogenic signaling triggered by secreted HMGA1, we conducted phosphoproteomic analysis, identifying hyperphosphorylation of Nucleophosmin 1 (NPM1), as a pivotal event that further amplifies tumor cell proliferation. Collectively, our findings reveal that a panel of chemotherapeutic agents stimulate a novel mutp53-dependent CK2-HMGA1-NPM1 axis that fuels PDAC proliferation in an autocrine/paracrine manner. Targeting this pathway at multiple levels emerges as a promising therapeutic strategy to counteract mutp53-driven tumor progression and improve patient outcomes.

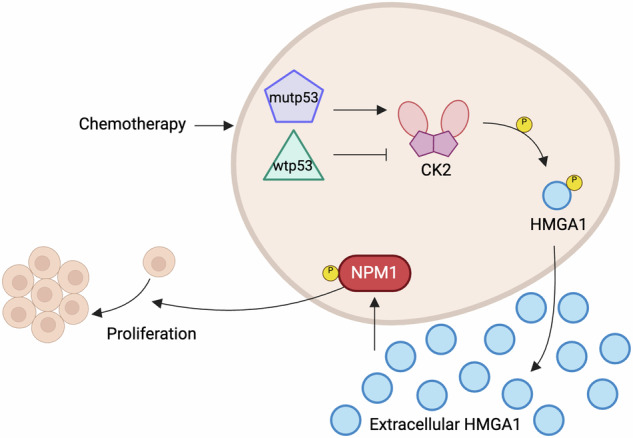

## Introduction

Pancreatic ductal adenocarcinoma (PDAC), one of the deadliest cancers, currently ranks as the third leading cause of cancer-related deaths and is projected to become the second by 2040, only surpassed by lung cancer [1, 2]. PDAC often goes undetected until late in progression, typically after distant metastasis, due to its silent progression or unspecific symptomatology, absence of early detection markers, and the pancreas’ hidden location, resulting in limited treatment options and alarmingly high mortality rates [3, 4]. Previous studies have consistently highlighted *KRAS*, *TP53*, *SMAD4*, and *CDKN2A* as the most frequently mutated genes in PDAC, marking them as key disease drivers [5–8]. Notably, *TP53* mutations occur in 50–70% of PDAC cases, playing a pivotal role in advancing the disease [9, 10]. These mutations often lead to a gain-of-function (GOF) activity, which drives tumor progression, fuels metastasis, and fosters resistance to chemotherapy [11]. Most of these mutations (80%) are missense, mainly occurring in the DNA-binding domain of the *TP53* gene with six “hotspot” amino acids most affected (R175, G245, R248, R249, R273, and R282) [12–15]. Current treatment options for PDAC are largely ineffective, providing only limited benefits for most patients [16]. This highlights the urgent need to develop new therapeutic strategies that can effectively target the unique biology of PDAC. Since mutant p53 (mutp53) reshapes the tumor microenvironment (TME) [12, 17], a promising approach may be to define the mutp53-driven secretome, the collection of factors secreted by tumor cells harboring mutp53, as a mean to identify new druggable targets that enhance cancer progression, immune evasion, drugs resistance and metastasis [18, 19].

Here, we first identify HMGA1 as clinically relevant protein within the mutp53-driven secretome. Then, we uncover the mechanisms that drive its secretion, highlighting the impact of standard chemotherapy on this process. Finally, we identify the phosphorylation events triggered by secreted HMGA1, thus highlighting both the potential paracrine effect of the secreted protein on adjacent cells and potential therapeutic targets within this pathway.

## Results

### Mutp53 promotes HMGA1 secretion in PDAC cells

**Fig. 1:**
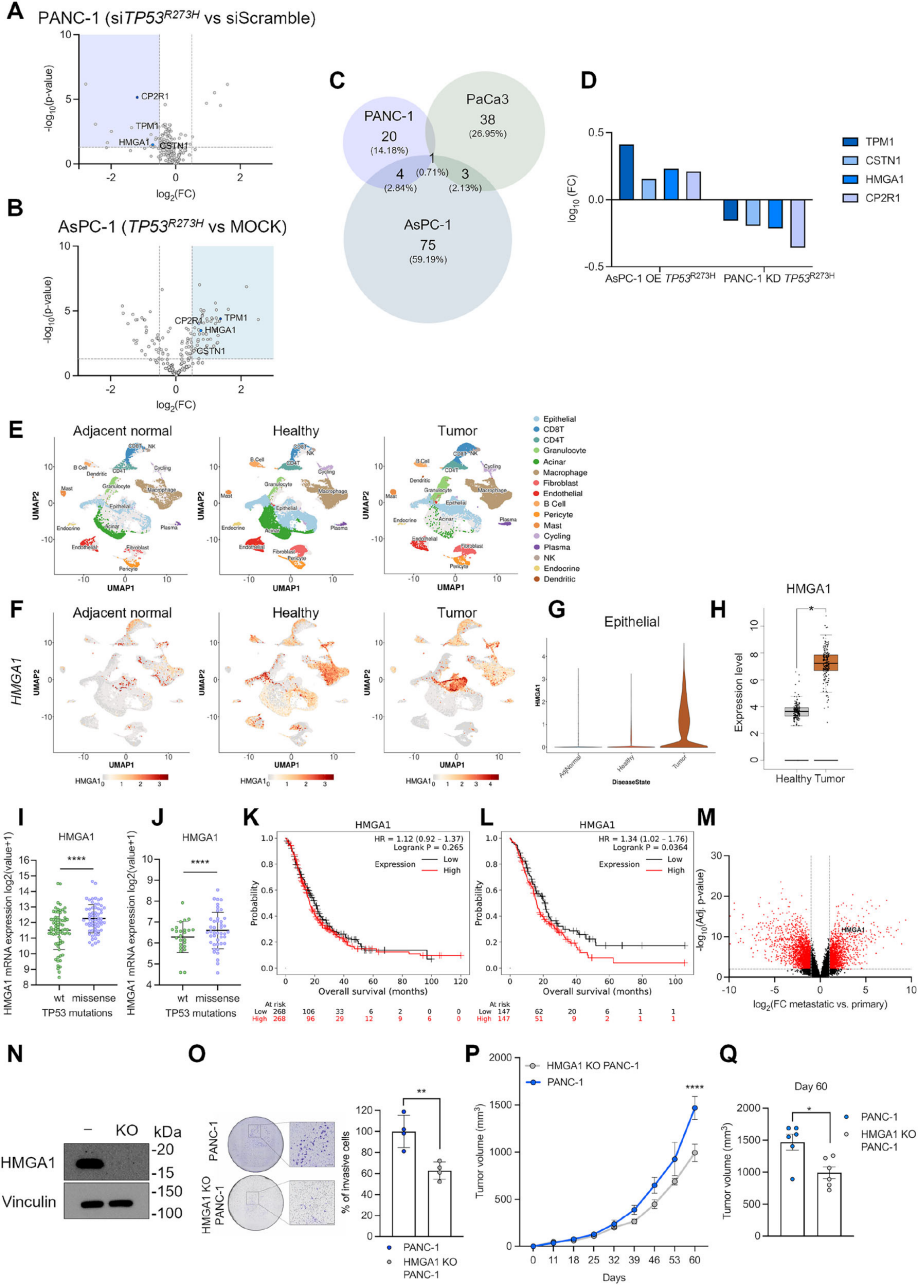
MS analysis of mutp53-dependent secreted proteins in human PDAC cells. **A** Differentially secreted proteins detected by MS analysis in the CM of PANC-1 cells transiently transfected with si*TP53*^*R273H*^ or siScramble. The 25 proteins significantly downsecreted are indicated by the light lavender-shaded rectangle underlaid on the plot. Vertical dashed lines indicate log2 fold change = ±0.5. Horizontal dashed line indicates the cut-off p-value *p* = 0.05. TPM1, CSTN1, HMGA1 and CP2R1 proteins are marked on the plot. **B** Differentially secreted proteins detected by MS analysis in the CM of AsPC-1 cells transiently transfected with *TP53*^*R273H*^ or MOCK plasmid. The 83 proteins significantly hypersecreted are indicated by the light teal-shaded rectangle underlaid on the plot. Among them, TPM1, CSTN1, HMGA1 and CP2R1 proteins are specifically highlighted. Vertical dashed lines indicate log2 fold change = ±0.5. Horizontal dashed line indicates the cut-off p-value *p* = 0.05. **C** Venn diagram indicating the overlaps of the differentially mut or wt p53-dependent secreted proteins detected by MS analysis. **D** Histogram showing 4 proteins (Tropomyosin 1 (TPM1), Calsyntenin 1 (CSTN1), High Mobility Group A1 (HMGA1), Cytochrome P450 Family 2 Subfamily R Member 1 (CP2R1)) hypersecreted by AsPC-1 (p53-null) cells overexpressing *TP53*^*R273H*^ and downsecreted by PANC-1 cells after KD of the same hot-spot mutp53 isoform. **E** UMAP visualization of all identified cell types present in the pancreatic microenvironment subset by disease state: Adjacent Normal (*n* = 3), Healthy (*n* = 6) and Tumor (*n* = 16). Data source: Pancreatic Tissue Single Cell Atlas. **F** UMAP visualizations showing *HMGA1* expression across the major cell populations subset by disease state (Adjacent Normal, Healthy, Tumor). Data source: Pancreatic Tissue Single Cell Atlas. **G** *HMGA1* gene expression level in tumor derived epithelial cells compared to adjacent normal or healthy epithelial cells (number of cells: 892 Adj.Normal; 14,380 Healthy; 9484 Tumor). Data source: Pancreatic Tissue Single Cell Atlas. **H** *HMGA1* gene expression level in tumor compared to normal tissue. Data source: GEPIA database. * p < 0.01. **I** *HMGA1* expression correlates with the mutational status of *TP53* (*n* = 66 no-mutation, *n* = 62 missense mutations). Data source: cBioportal database, *TCGA PanCancer Atlas*. (Wilcoxon test). **** p < 0.0001. **J** *HMGA1* expression level in PDAC patient with *TP53*^*mut*^ (*n* = 24 no-mutation, *n* = 43 missense mutations Data source: cBioportal database, *QCMG Nature 2016*. (Wilcoxon test). **** p < 0.0001. **K** Kaplan-Meier (KM) plot of survival probability (log-rank test) for PDAC patients only as obtained from KM-plotter database using the default parameters. **L** Kaplan-Meier survival plot after PDAC patients’ stratification for tumor stage (S2, S3, S4) in KM plotter database showing *HMGA1* expression is a prognostic factor in advanced pancreatic cancer. **M** Volcano plot of differential gene expression (DGE) analysis for metastatic vs primary tumors using the microarray dataset GSE71729 showing *HMGA1* is significantly highly expressed in metastatic patients (Log2FC = 2.383837; −log10(Adj. p-value) = 10.47756). Red highlights indicate significant regulated genes; black highlights indicate non-significant genes. Vertical dashed lines indicate log2 fold change = ±1. Horizontal dashed line indicates *p* = 0.01. **N** Immunoblot validation of HMGA1 KO in human PANC-1 cell line. KO denotes HMGA1 KO cells, while the minus sign (-) represents the parental cells. Vinculin was used as a loading control. **O** Representative images, with corresponding magnifications, of PANC-1 (top) and HMGA1-KO PANC-1 (bottom) cells invading through Matrigel-coated transwell inserts (8 μm pore size) after 24 h of incubation at 37 °C. Right panel: bar plot quantification of the percentage of invasive cells. Data are presented as mean ± SD (n = 4). (Unpaired t-test). **p < 0.01. **P** Tumor volume (mm^3^) from subcutaneous injection of either PANC-1 or HMGA1 KO PANC-1 cells in immunodeficient mice. Data plotted are mean tumor volumes + SEM (*n* = 6 for each cohort). (Two-way ANOVA). ****p < 0.0001. **Q** Individual tumor volumes + SEM at the endpoint (Unpaired t-test). *p < 0.05.

To identify mutp53-dependent secreted proteins by human PDAC cells, we performed high-resolution sequential window acquisition of all theoretical mass spectra (SWATH-MS) analysis on the conditioned media (CM) collected from various PDAC cell lines upon genetic manipulation of *TP53* gene. First, since the mass spectrometry (MS) analysis of the secretome requires a serum-free medium, 22 h before CM collection the medium was replaced with medium without FBS and cell viability was assessed to ensure that the serum-free conditions did not adversely affect the cells employed in this study (Supplementary Fig. S1A). This serum-free period of incubation has been chosen based on our previous work [20]. We conducted MS analysis on CM from PANC-1 cells following knockdown (KD) of the R273H hotspot mutant p53 isoform, which is endogenously expressed in this cell line (Supplementary Fig. S1B). This analysis revealed 25 proteins whose secretion was significantly downregulated in a mutp53-dependent manner (Fig. 1A and Table S1). Furthermore, we integrated these data with previously published datasets from our laboratory, which examined (i) p53-null AsPC-1 PDAC cells after exogenous overexpression (OE) of the mutp53 isoform *TP53*^*R273H*^ or MOCK control, identifying 83 mutp53-dependent proteins with significantly increased secretion (Fig. 1B) [20], and (ii) PaCa3 cells with silenced *TP53*^*WT*^, revealing 42 proteins significantly downregulated by *TP53*^*WT*^ loss [21] (Fig. 1C). To focus on the secretome alterations specifically driven by mutp53, we narrowed our study to four proteins that were hypersecreted upon *TP53*^*R273H*^ OE in AsPC-1 and downsecreted after *TP53*^*R273H*^ KD in PANC-1 but absent in the *TP53*^*WT*^-secretome (Fig. 1D). For all the MS analyses, we set significance thresholds with log2 fold change values above +0.5 or below -0.5, alongside p-values lower than 0.05.

The four proteins are Tropomyosin 1 (TPM1), Calsyntenin 1 (CSTN1), Cytochrome P450 Family 2 Subfamily R Member 1 (CP2R1), and High Mobility Group A1 (HMGA1).

To further identify the most clinically relevant targets in PDAC patients, we analyzed their gene expression profile (homonymous gene *TPM1*, *CLSTN1*, *CYP2R1*, *HMGA1*) using the single-cell RNA sequencing (scRNA-seq) data from the Pancreatic Tissue Single Cell Atlas [22, 23]. First, using the two-dimensional uniform manifold approximation and projection (UMAP), we visualized all identified cell types present in the pancreatic microenvironment categorized by disease state: adjacent normal (*n* = 3; *cells* = 7,583), healthy (*n* = 6; *cells* = 44,068) and tumor (*n* = 16, across different disease stages; *cells* = 41,277) (Fig. 1E and Supplementary Fig. S1C). Then, we matched the expression patterns of the four genes across all the previously identified cell populations (Fig. 1F and Supplementary Fig. S1D, F, H), with a specific focus on epithelial cells. *TPM1* showed higher expression in epithelial cells from adjacent normal compared to those from healthy tissues and tumors (Supplementary Fig. S1E). *CLSTN1* was expressed in both tumor and healthy epithelial cells (Supplementary Fig. S1G), while *CYP2R1* had almost undetectable expression (Supplementary Fig. S1I). In contrast, *HMGA1* was significantly more expressed in tumor-derived epithelial cells compared to their healthy counterparts (Fig. 1G). HMGA1 has previously been reported to affect the polarization state and recruitment of macrophages in the tumor microenvironment (TME). Thus, the expression of HMGA1 by tumor derived epithelial cells may influence the tumor immune microenvironment (TIME). Interestingly, our analysis revealed the increased expression of HMGA1 within macrophages themselves, which other groups suggest may fine-tune the expression of pro-inflammatory genes [24, 25]. This pattern of *HMGA1* overexpression in cancer cells was further validated using another publicly available dataset, strengthening its relevance as a target for further studies (Fig. 1H). Thus, HMGA1 emerged as a mutp53-dependent secreted protein with distinct and elevated expression in tumor cells compared to adjacent normal or healthy tissues, making it the prime candidate for further study.

HMGA1 is an architectural transcription factor essential for remodeling chromatin structure and regulating transcription factor-DNA interactions [26, 27]. It has a crucial role in cell transformation and its elevated expression is consistently observed in various human malignant neoplasias [26]. Overexpression of *HMGA1* in both human and murine cancer cells has been shown to enhance colony formation, as well as promote cancer cell invasion and metastasis, making it a negative prognostic modulator [28]. Conversely, HMGA1 depletion not only suppresses cancer cell proliferation but also triggers programmed cell death, increasing the sensitivity of cancer cells to chemotherapy [29]. To determine if *HMGA1* expression is specifically influenced by the presence of *TP53* mutations, we investigated whether there is a correlation between *HMGA1* mRNA expression levels and the mutational status of *TP53*. By conducting this analysis in two independent datasets, we consistently found that *HMGA1* is significantly more expressed in PDAC patients harboring *TP53* missense mutations, highlighting its potential as a therapeutic target for PDAC (Fig. 1I, J). Notably, we found that *HMGA1* expression did not correlate with overall survival in PDAC patients when analyzed as a whole, but after stratifying patients by tumor stage (as per AJCC cancer staging manual, VIII Edition; S2, S3, S4), HMGA1 emerges as a prognostic factor, particularly in late-staged PDAC, indicating its potential clinical relevance in later stages of the disease (Fig. 1K, L). Additionally, *HMGA1* is significantly more expressed in metastatic patients compared to non-metastatic ones (Fig. 1M), further supporting its clinical relevance in patients with late-staged. Our bioinformatics analysis clearly showed that HMGA1 represents a promising target in PDAC patients.

To further investigate HMGA1 function, we first generated a CRISPR/Cas9 HMGA1 knock-out (KO) PANC-1 cell line (Fig. 1N). Using this model, we demonstrated that HMGA1 deficiency markedly reduced the invasive capacity of PANC-1 cells in vitro (Fig. 1O) and significantly impairs tumor growth in vivo in a xenograft mouse model of pancreatic adenocarcinoma (Fig. 1P, Q). This result is consistent with previous findings showing that the absence of HMGA1 slows PDAC progression and metastasis [30]. However, this experimental setting did not allow us to distinguish between the roles of intracellular and secreted HMGA1, whose role in the PDAC TME is still unknown.

### Mutp53-dependent HMGA1 secretion promotes tumor proliferation

**Fig. 2:**
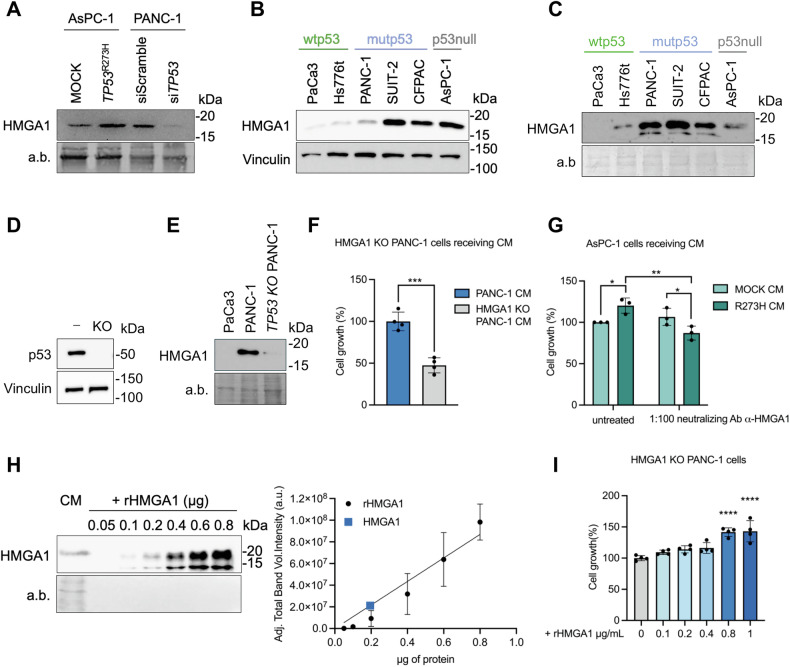
Mutp53 drives tumor proliferation through the secretion of HMGA1. **A** Immunoblot of secreted HMGA1 protein validating the MS-data. Amido black staining (a.b.) was used as loading control for WB. **B** Immunoblot of HMGA1 protein in different human PDAC cell lines. **C** Immunoblot of secreted HMGA1 protein in different human PDAC cell lines. **D** Immunoblot validation of *TP53* KO in human PANC-1 cell line. KO denotes *TP53* KO cells, while the minus sign (−) represents the parental cells. Vinculin was used as a loading control. **E** Immunoblot of secreted HMGA1 protein in PaCa3, PANC-1 and *TP53* KO PANC-1 cells. **F** Cell growth percentage measured by cristal violet assay in HMGA1 KO PANC-1 cells cultured for 48 h with PANC-1 CM or HMGA1 KO PANC-1 CM. (Unpaired t-test). *** p < 0.001. **G** Cell growth percentage measured by cristal violet assay in p53-null AsPC-1 cells cultured for 48 h with R273H CM or MOCK CM after the addition of anti-HMGA1 antibody (0.226 µg/µl, 1:100 in growth medium) or the IgG Isotype Control (Cell signaling, 5742). (Two-way ANOVA). *p < 0.05, **p < 0.01. **H** Immunoblot analysis of HMGA1 protein in PANC-1 CM alongside different µg of rHMGA1 protein. On the right, the slope obtained from linear regression analysis of the average adjusted total band intensity values + SD (arbitrary units, a.u.) of immunoreactivity corresponding to rHMGA1. The adjusted total band intensity values were derived from densitometric analysis of the immunoreactive bands for HMGA1 secreted by PANC-1 cells, as well as rHMGA1 used as a standard with increasing sample loads. Data were analyzed using Image Lab Software (Bio-Rad, version 6.1.0 build 7). The blue square on the slope represents the value corresponding to the secreted HMGA1. **I** Cell growth percentage measured by cristal violet assay in HMGA1 KO PANC-1 cells cultured for 72 h after treatment with different doses of rHMGA1 protein. (One-way ANOVA). ****p < 0.0001.

To uncover HMGA1’s extracellular role, we first validated our MS findings via western blot (WB). The immunoblot analysis of HMGA1 in the protein extracts from the CM confirmed that HMGA1 is hypersecreted when mutp53 is expressed (Fig. 2A), whereas the control at the proteome level confirmed the successful OE and KD of mutp53 in AsPC-1 and PANC-1 cells, respectively (Supplementary Fig. S1J). At this point, we extended our study to other human PDAC cell lines. After assessing cell viability to ensure that the cells remained viable after the 22 h in serum-free conditions (Supplementary Fig. S1K), we showed that all cell lines expressed HMGA1. Notably, cell lines derived from metastatic lesions (SUIT-2, CFPAC and AsPC-1) exhibited higher levels of HMGA1 in their proteomes (Fig. 2B) compared to those derived from primary tumors, consistent with our bioinformatics analysis (Fig. 1M). Interestingly, the expression level did not correlate with protein secretion; indeed, we demonstrated that HMGA1 hypersecretion correlates with human PDAC cells harboring mutations in the *TP53* gene (i.e., PANC-1, SUIT-2, CFPAC) (Fig. 2C). To further investigate the role of p53 in this context, we used a CRISPR/Cas9 *TP53* KO PANC-1 cell line [31] (Fig. 2D) and confirmed that mutp53 is essential for driving HMGA1 secretion (Fig. 2E). Building on these findings, we next sought to define HMGA1’s function once in the extracellular space. To this end, we assessed the growth of HMGA1 KO PANC-1 cells after 48 h of culture in either PANC-1 CM or HMGA1 KO PANC-1 CM. Cells subjected to the absence of extracellular HMGA1 exhibited reduced proliferation (Fig. 2F). Similarly, we evaluated the growth of p53-null AsPC-1 cells after 48 h of culture in CM derived from AsPC-1 cells overexpressing *TP53*^*R273H*^ or the control (MOCK). This analysis confirmed that the mutp53-driven secretome exerts hyperproliferative effects [20]. Notably, we demonstrated that neutralizing extracellular HMGA1 with an anti-HMGA1 antibody reversed this growth effect (Fig. 2G). To further investigate the role of extracellular HMGA1 in tumor proliferation, we aimed to quantify HMGA1 in the CM. Due to the ineffectiveness of the commercially available ELISA kits, we produced a recombinant human HMGA1 protein (rHMGA1) and conducted a Western blot analysis [32] to estimate the amount of HMGA1 secreted by PANC-1 cells. Based on linear regression analysis of immunoreactive band intensities for secreted HMGA1 and rHMGA1 as a standard, we estimated that ~0.2 µg of HMGA1 was secreted in the CM of about 3 × 105 PANC-1 cells (Fig. 2H). Next, we assessed the growth of HMGA1 KO PANC-1 cells after 72 h of culture with different concentrations of rHMGA1. The results confirmed that the presence of HMGA1 in the medium alone was sufficient to induce hyperproliferation (Fig. 2I), as previously observed with the CM.

### Anti-cancer treatments boost HMGA1 secretion in mutp53-expressing cells

**Fig. 3:**
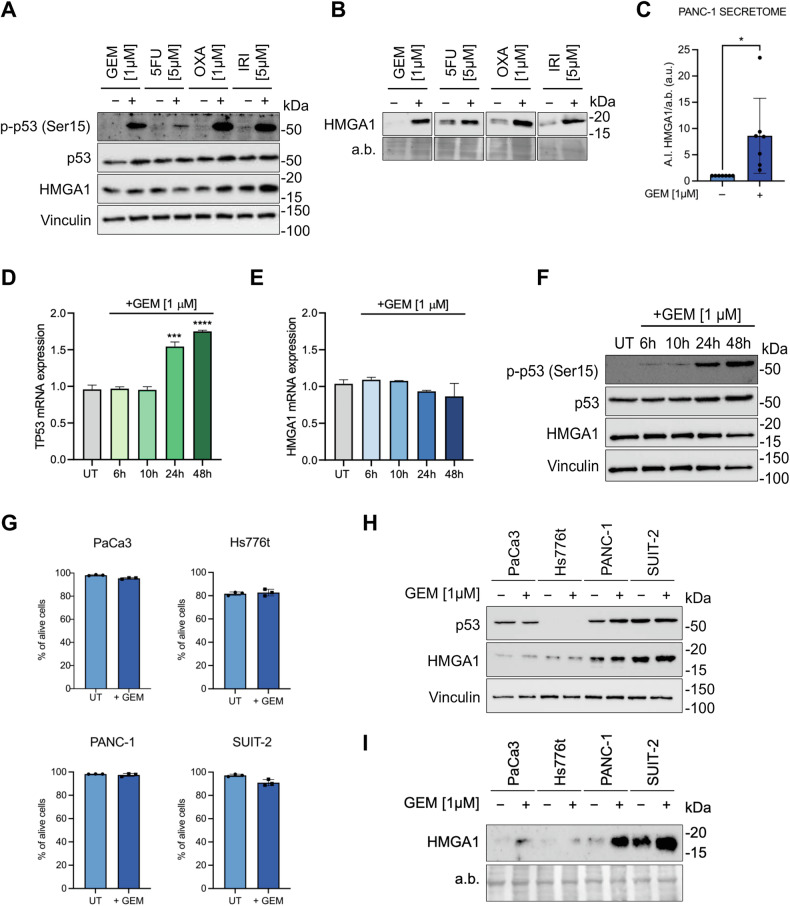
Chemotherapeutic drugs promotes HMGA1 hypersecretion in mutp53 PDAC cells. **A** Immunoblot of p-p53, p53 and HMGA1 proteins in PANC-1 cells after treatment with different anti-cancer drugs at sublethal doses (1 µM gemcitabine (GEM), 5 µM 5-fluorouracil (5-FU), 1 µM oxaliplatin (OXA) and 5 µM irinotecan (IRI)). **B** Immunoblot of HMGA1 protein in PANC-1 cells secretome after treatment with different anti-cancer drugs at sublethal doses. **C** Bar charts depict A.I. (a.u.) of HMGA1 secreted by PANC-1 cells with or without 1 µM GEM treatment versus a.b. analyzed using Image Lab Software (Bio-Rad, version 6.1.0 build 7). Data plotted are mean of seven independent experiments ± SD. (Unpaired t-test). *p < 0.05. **D** qPCR showing *TP53* expression upon sublethal dose GEM treatment of PANC-1 cells for 6, 10, 24 and 48 h. ***p < 0.001, ****p < 0.0001. **E** qPCR showing *HMGA1* expression upon sublethal dose of GEM treatment of PANC-1 cells for 6, 10, 24, and 48 h. **F** Immunoblot of p-p53, p53, and HMGA1 proteins in PANC-1 cells upon sublethal dose GEM treatment for 6, 10, 24 and 48 h. **G** Bar charts of PaCa3, Hs776t, PANC-1 and SUIT-2 cells viability (PI-/Ann V-) after 24 h treatment with 1 µM GEM. **H** Immunoblot of p53 and HMGA1 proteins in two cell lines carrying *TP53*^*w*t^ (PaCa3 and Hs776t) and two cell lines harboring *TP53*^*R273H*^ (PANC-1 and SUIT-2) after being treated or not with 1 µM of GEM. **I** Immunoblot of HMGA1 in human PDAC cells secretome after 1 µM GEM treatment.

Chemotherapy is the primary treatment for PDAC [3, 16], and chemoresistance is induced by mutp53 in PDAC patients [12, 33]. Thus, we aimed to investigate how chemotherapy impacts the mutp53-driven secretion of HMGA1. We initially screened various concentrations of clinically relevant chemotherapeutic drugs for the treatment of PDAC; i.e., gemcitabine (GEM) and three chemotherapeutics included in FOLFIRINOX: 5-fluorouracil (5-FU), oxaliplatin (OXA), and irinotecan (IRI). We determined the highest dose that preserved PANC-1 cell viability, defined as ≥90% viable cells by the end of treatment, thus ensuring minimal direct toxicity (Supplementary Fig. S2A). After determining the optimal sublethal dose for each drug (1 µM GEM, 5 µM 5-FU, 1 µM OXA, and 5 µM IRI), we further confirmed the ≥90% of cell viability cut off also using flow cytometry (Annexin V^-^/PI^-^ cells) (Supplementary Fig. S2B). We therefore evaluated the effect of the chemotherapeutic treatment on HMGA1 protein expression and secretion. At intracellular proteome level, HMGA1 expression is unaffected (Fig. 3A), while we detected a massive hypersecretion of HMGA1 in PANC-1 cells after 24 h of chemotherapeutic treatments (Fig. 3B). Each of the chemotherapeutic treatments promoted p53 Ser15 phosphorylation (Fig. 3A), a key activation site [34], suggesting that chemotherapy may abnormally stimulate mutp53 [35], thereby enhancing HMGA1 secretion.

Since GEM is the chemotherapy that most significantly drives HMGA1 hypersecretion (Fig. 3C and Supplementary Fig. S2C), we selected it to investigate the underlying mechanism. First, we assessed HMGA1 mRNA expression and protein levels across different GEM treatments. Our findings revealed an increased transcription, translation and phosphorylation of mutp53, confirming the chemotherapy-driven activation of mutp53. However, we did not observe an increase in either HMGA1 transcription or translation, indicating that the GEM-induced HMGA1 hypersecretion is likely driven by the activation of mutp53 rather than by increased synthesis of HMGA1 (Fig. 3D, E, F). To extend our findings, we used GEM to treat other human PDAC cell lines. After verifying that all these cell lines remained viable post-treatment (Fig. 3G), we observed that also in this experimental setting intracellular HMGA1 protein levels remained unchanged after treatment (Fig. 3H). Notably, GEM induces HMGA1 hypersecretion exclusively in cell lines harboring *TP53* mutations (i.e., PANC-1 and SUIT-2) (Fig. 3I), underscoring the essential role of mutp53 in driving HMGA1 secretion.

### Gemcitabine enhances CK2-mediated HMGA1 secretion in cells harboring mutant p53

HMGA1 is secreted in invasive triple-negative breast cancer (TNBC) cells via unconventional protein secretion (UPS) orchestrated by Casein kinase 2 (CK2), bypassing the classical endoplasmic reticulum (ER)-Golgi secretory pathway [36]. CK2 is a known regulator of UPS [37–39] and it has been previously shown to play a role in regulating HMGA1 secretion [36]. Namely, the C-terminal tail of HMGA1—a region associated with cancer aggressiveness [40]—is phosphorylated by CK2 [41]. Given the involvement of other kinases in HMGA1 phosphorylation, we first sought to identify the most clinically relevant kinase in PDAC patients. To this end, we analyzed the gene expression profiles of key candidate kinases (*CSNK2A1*, *CDK1*, *CDK2*, *HIPK2*) using scRNA-seq data from the Pancreatic Tissue Single Cell Atlas [22, 23]. Using UMAP, we visualized all identified cell types within the pancreatic microenvironment, categorized by disease state: adjacent normal (n = 3; cells = 7583), healthy (n = 6; cells = 44,068), and tumor (n = 16; cells = 41,277 across different disease stages) (Supplementary Fig. S3A). We then examined the expression patterns of the four kinase genes across all identified cell populations (Supplementary Fig. S3C, E, G, I), with a specific focus on epithelial cells (Supplementary Fig. S3D, F, H, J). Among the analyzed genes, only *CSNK2A1*, which encodes the catalytic subunit of CK2, exhibited significantly higher expression in tumor-derived epithelial cells compared to healthy or adjacent normal epithelial cells (Supplementary Fig. S3C, D). This observation is further supported by the heatmap of kinase expression across epithelial cell populations (Supplementary Fig. S3B). At this point to further identify the leading kinase phosphorylating the most frequent HMGA1 phosphorylation sites, we queried the PhosphoSitePlus® (v6.8.0) database to assess the most frequently detected post-translational modification (PTM) sites on HMGA1 identified by mass spectrometry. Among them, the most frequently observed phosphorylation sites were S102-p and S103-p, which are known targets of CK2, providing further support for its role in regulating HMGA1 (Supplementary Fig. S3K). Together, these data support the biological and mechanistic relevance of CK2 in regulating HMGA1 function and secretion in the context of PDAC.

**Fig. 4:**
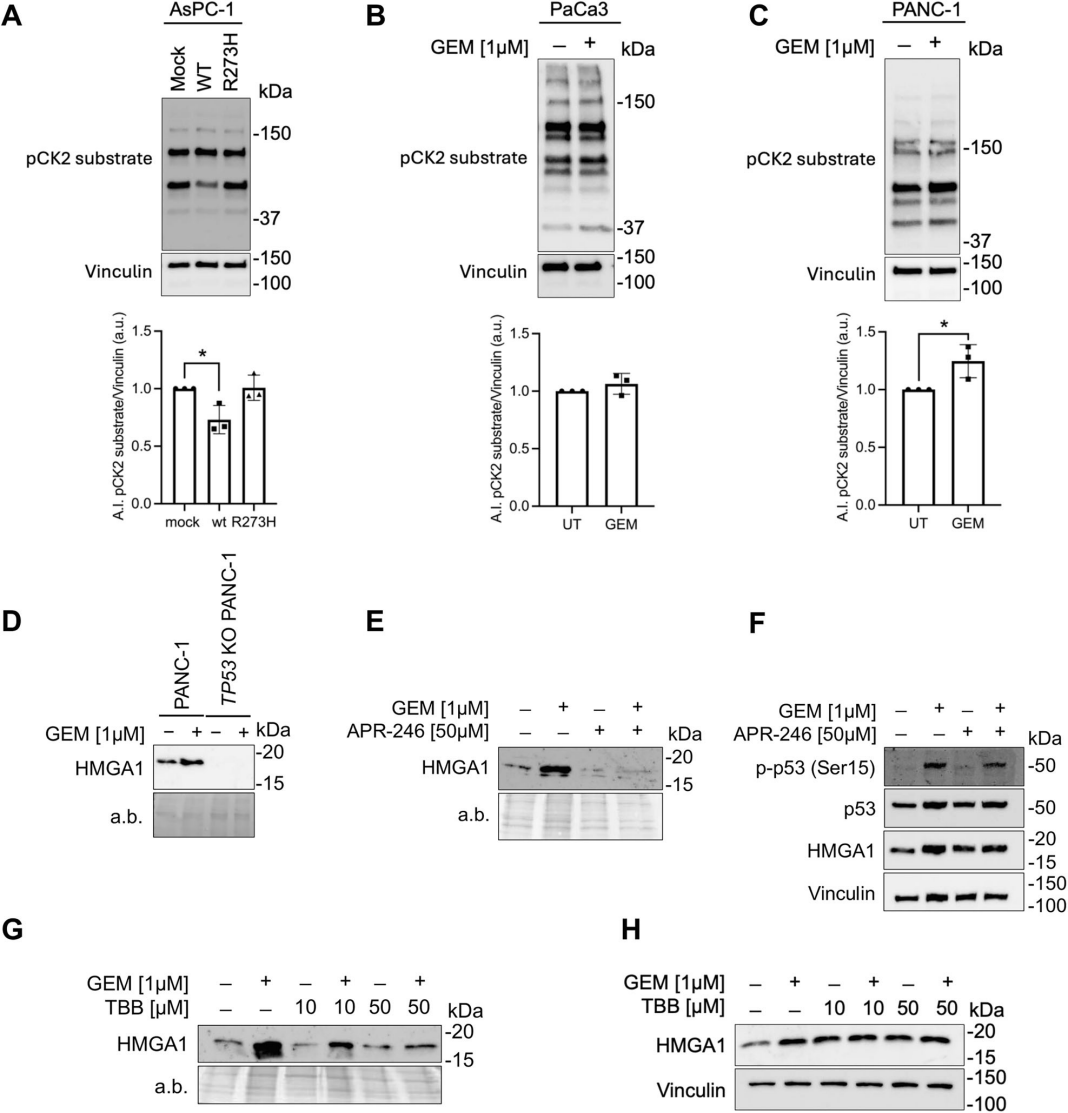
Opposing roles of wild-type and mutant p53 in CK2-mediated HMGA1 secretion. **A** Immunoblot of CK2-phosphorylated substrate (pCK2 substrate) 48 h after AsPC-1 cells were transiently transfected with MOCK, *TP53*^*WT*^ or *TP53*^*R273H*^. Bar charts depict the average signal intensity (A.I.) in a.u. of phospho-CK2 substrate versus Vinculin. Data plotted are mean of three independent experiments +/- SD. (One-way ANOVA). * p < 0.05. **B** Immunoblot of pCK2 substrate 24 h after 1 µM GEM treatment of PaCa3 cells. Bar charts depict the A.I. of pCK2 substrate versus Vinculin (a.u.). Data are presented as the mean of three independent experiments +/- SD. (Unpaired t-test). **C** Immunoblot of pCK2 substrate 24 h after 1 µM GEM treatment of PANC-1 cells. Bar charts depict the A.I. of pCK2 substrate versus Vinculin (a.u.). Data plotted are mean of three independent experiments ± SD. (Unpaired t-test). *p < 0.05. **D** Immunoblot of HMGA1 protein in PANC-1 and *TP53* KO PANC-1 secretome after treatment with 1 µM GEM. **E** Immunoblot of HMGA1 in PANC-1 cell secretome after 1 µM GEM and 50 µM APR-246 treatments. **F** Immunoblot of p-p53, p53 and HMGA1 proteins in PANC-1 cells treated with or without 1 µM GEM and with or without 50 µM APR-246. **G** Immunoblot of HMGA1 in PANC-1 cell secretome after 1 µM GEM and 10 µM or 50 µM TBB treatments. **H** Immunoblot of HMGA1 proteins in PANC-1 cells treated with or without 1 µM GEM and with or without 10 or 50 µM TBB.

To explore the role of WT and mutp53 in regulating CK2 activity, we measured CK2-phosphorylated substrates (pCK2 substrate) by western blot analysis. Our results indicate a decrease in pCK2 substrate levels when *TP53*^*WT*^ is overexpressed in AsPC-1 cells, compared to cells having mutp53 (R275H) or lacking p53 (MOCK) (Fig. 4A), suggesting a link between p53 status and CK2 activity. To unravel this potential link, we analyzed the effect of GEM treatment, known to activate mutp53 [35], on CK2 activity across different human PDAC cell lines. Interestingly, GEM treatment did not alter CK2 activity in PaCa3 cells carrying *TP53*^*WT*^ (Fig. 4B), whereas it led to increased pCK2 substrate levels in PANC-1 cells expressing *TP53*^*R273H*^ (Fig. 4C). Given prior evidence that wtp53 can inhibit CK2 activity [42], these findings suggest that wild-type and mutant p53 might exert opposing effects on CK2, with wtp53 acting as an inhibitor and mutp53 as an activator, whose function is further enhanced by chemotherapy. To better define this aspect, we also showed that GEM-treatment of *TP53* KO PANC-1 cells (Fig. 4D) did not result in HMGA1 hypersecretion, strongly confirming that mutp53 is necessary for chemotherapy-induced HMGA1 release.

To further clarify the opposing effects of wild-type and mutant p53 on HMGA1 secretion, we treated PANC-1 cells with a sublethal yet functionally effective dose (Supplementary Fig. S4A, B, C) of APR-246 (also known as Eprenetapopt or PRIMA-1^MET^), a small molecule known to restore wtp53 function in missense mutp53 expressing cells. Notably, restoring the wtp53 conformation to mutp53 fully abrogated the HMGA1 secretion, even when cells were stimulated with GEM (Fig. 4E) at a concentration that typically induces Ser15 phosphorylation and activates p53 (Fig. 4F). Overall, these findings suggest that the restored wtp53 function through APR-246 can counteract the GEM-induced hypersecretion of HMGA1, reinforcing the regulatory role of functional p53 in modulating CK2 activity and, consequently, HMGA1 secretion. To confirm CK2 involvement in HMGA1 secretion, we treated PANC-1 cells with increasing non-toxic but functional concentrations of the CK2 inhibitor 4,5,6,7-tetrabromobenzotriazole (TBB) (Supplementary Fig. S4D, E, F). This treatment resulted in a dose-dependent decrease of HMGA1 secretion, even in presence of GEM stimulation (Fig.4G, without notable changes in HMGA1 levels within the proteome (Fig. 4H and Supplementary Fig. S4G). Altogether, these data demonstrate that HMGA1 secretion triggered by chemotherapy is mediated by the mutp53-CK2 axis and that HMGA1 secretion can limit the antiproliferative effects of chemotherapeutic drugs.

### HMGA1-dependent phosphorylation of NPM1 promotes tumor proliferation

**Fig. 5:**
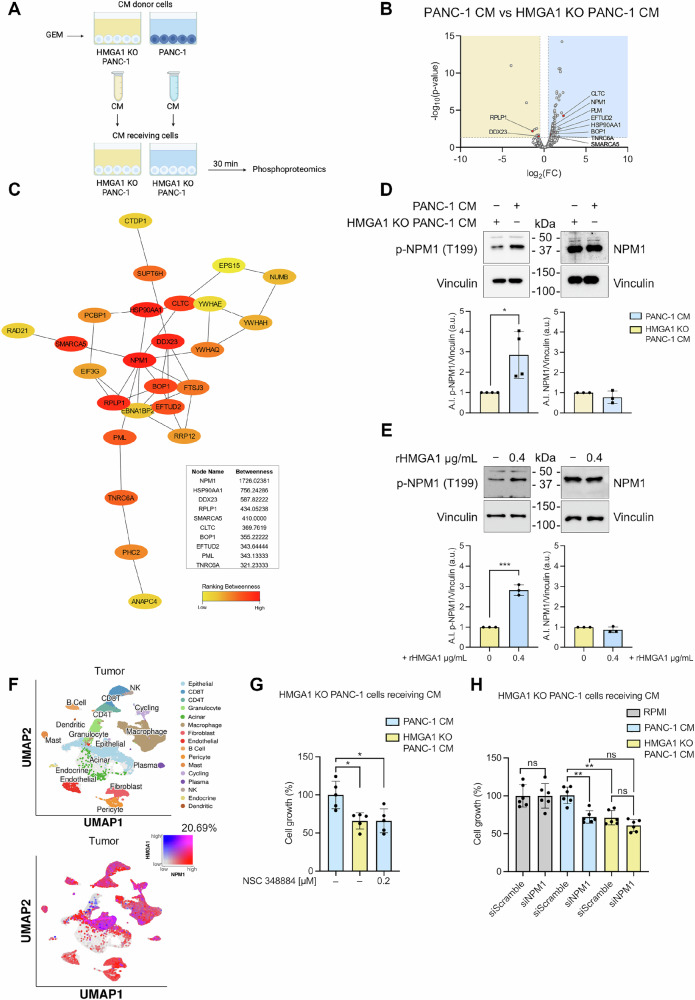
Phosphoproteomic profiling uncovers the HMGA1-NPM1 axis as a crucial driver of tumorigenesis. **A** Schematic workflow of the phosphoproteomics experiment. Created with BioRender.com. **B** Volcano plot of phosphoproteomics analysis on HMGA1 KO PANC-1 cells treated either with HMGA1 KO PANC-1 CM or PANC-1 CM for 30 min, plotting log2 fold change PANC-1 CM vs HMGA1 KO PANC-1 CM treated. The 90 phosphoproteins significantly upregulated are indicated by the light blue-shaded rectangle underlaid on the plot. The 11 downregulated phosphoproteins are indicated by the light yellow-shaded rectangle underlaid on the plot. Vertical dashed lines indicate log2 fold change = ±0.5. Horizontal dashed line indicates *p* = 0.05. Red highlights indicate the top 10 significant up- and down- regulated phosphorylated proteins identified as hubs according to the betweenness criterium. **C** Subnetwork of top 25 hub genes from protein-protein interaction network. Genes were ranked by betweenness centrality mode. The node color reflects the degree of connectivity through a color scale ranging from red (high) to yellow (low). In the table the top 10 hub genes with the node name and the betweenness score. **D** Immunoblots of p-NPM1 and NPM1 3 h after PANC-1 CM or HMGA1 KO PANC-1 CM treatment of HMGA1 KO PANC-1 cells. Bar charts depict the A.I. of p-NPM1 versus Vinculin (on the left) and NPM1 versus Vinculin (on the right, a.u.). Data plotted are mean of four independent experiments +/- SD for p-NPM1 and three independent experiments ± SD for NPM1. (Unpaired t-test). * p < 0.05. **E** Immunoblots of p-NPM1 and NPM1 1 h after treatment of HMGA1 KO PANC-1 cells with rHMGA1 protein (0.4 µg/mL). Bar charts depict the A.I. of p-NPM1 versus Vinculin (on the left) and NPM1 versus Vinculin (on the right, a.u.). Data plotted are mean of three independent experiments ± SD. (Unpaired t-test). *** p < 0.001. **F** UMAP visualization of all identified cell types present in tumor pancreatic microenvironment displaying the (20.69%) co-expression of NPM1 and HMGA1 genes (number of cells: 18754 NPM1; 1447 HMGA1; 8540 Both; 12536 None). Data source: Pancreatic Tissue Single Cell Atlas. **G** Cell growth percentage measured by cristal violet assay in HMGA1 KO PANC-1 cells cultured for 24 h with CM-PANC-1, CM-HMGA1 KO PANC-1 or CM-PANC-1 + 0.2 µM NSC 348884. (Two-way ANOVA). *p < 0.05. **H** Cell growth percentage measured by crystal violet assay in HMGA1 KO PANC-1 cells transfected with siScramble or siNPM1. After 24 h, cells were replated in 96-well plates. 24 h later, cells were treated for an additional 24 h with RPMI, PANC-1 CM, or HMGA1 KO PANC-1 CM. (Two-way ANOVA). **p < 0.01.

Since the oncogenic effects of mutp53 have been shown to involve the modulation of the tumor secretome, in an autocrine or paracrine manner [43], we hypothesized that HMGA1, once secreted, may stimulate pro-growth signaling in cancer cells. To investigate the downstream effects of secreted HMGA1 on PDAC cells, we first induced HMGA1 hypersecretion with GEM treatment for 24 h, followed by extensive washing and addition of fresh media. After 22 h, we collected the CM from both HMGA1 KO PANC-1 and PANC-1 cells. Subsequently, we treated HMGA1 KO PANC-1 cells with each type of CM for 30 min (Fig. 5A) and then, performed a phosphoproteomic analysis. We identified 90 significantly upregulated and 11 downregulated phosphoproteins (Fig. 5B and Table S2), shedding light on the specific phosphorylation changes driven by extracellular HMGA1 and their possible contribution to tumor aggressiveness. We conducted a STRING analysis of the 101 significant phosphoproteins to map protein-protein interactions within this HMGA1-modulated phosphorylation landscape (Supplementary Fig. S5A), and we analyzed the STRING interaction results using the CytoHubba plugin in Cytoscape to identify hub genes within the phosphorylation network (Supplementary Fig. S5B). Among the top 25 hub genes identified, we focused on Nucleophosmin 1 (NPM1), which emerged as the highest-scoring hub marking it as a critical component within this network (Fig. 5C). NPM1 is a multifunctional phosphoprotein involved in essential cellular processes, such as ribosome biogenesis, cell proliferation, DNA repair, and apoptosis [44]. In PDAC, NPM1 has a role in tumor progression and chemotherapy resistance [45, 46]. We focused on Thr199 phosphorylation of NPM1 because it is specifically implicated in regulating NPM1’s functional activity, including its involvement in cell proliferation and tumor progression [47, 48]. WB analysis confirmed that extracellular HMGA1 indeed drives Thr199 hyperphosphorylation, while total NPM1 protein levels remain unchanged at the proteome level (Fig. 5D). Notably, the same result was reproduced using the rHMGA1 (Fig. 5E). To assess the clinical significance of NPM1, we analyzed its expression using scRNA-seq data from the Pancreatic Tissue Single Cell Atlas (Fig. 1E and Supplementary Fig. S6A). Then, by querying *NPM1* expression within the tumor microenvironment and comparing between adjacent normal, healthy and tumor tissues, we found that it is particularly expressed in tumor epithelial cells (Supplementary Fig. S6B, C). Furthermore, we examined *NPM1* and *HMGA1* co-expression in the tumor samples, revealing a notable 20.69% co-expression rate (Fig. 5F). These data underscore a potential relationship between NPM1 and HMGA1, highlighting their possible cooperative roles in the pathogenesis of pancreatic cancer and warranting further investigation into their functional interactions. To further support the association between HMGA1 and NPM1, we performed an mRNA co-expression analysis using two independently curated PDAC datasets (Pancreatic Adenocarcinoma, TCGA, GDC and Pancreatic Ductal Adenocarcinoma, CPTAC, Cell 2021) accessed through cBioPortal. This analysis revealed a statistically significant correlation between HMGA1 and NPM1 expression levels (Supplementary Fig. S6D, E). The consistent co-expression observed in scRNA-seq and bulk RNA-seq data suggests an association between the two genes, further strengthening the functional link revealed by our phosphoproteomic and network-based analyses.

To elucidate the functional role of p-NPM1, we utilized a specific NPM1 inhibitor, NSC 348884, previously applied in acute myeloid leukemia (AML) models [49]. After determining the highest dose that maintained PANC-1 cell viability (≥90% 24 h post-treatment) (Supplementary Fig. S6F), we treated HMGA1 KO PANC-1 cells with CM from PANC-1 cells, HMGA1 KO PANC-1 cells, or PANC-1 CM co-treated with the non-lethal dose of NSC348884. Notably, we observed that inhibiting NPM1 significantly reduced cell proliferation to a level comparable to that seen in conditions lacking extracellular HMGA1 (Fig. 5G and Fig. 2F).

This experiment was confirmed using a genetic loss-of-function strategy; HMGA1 KO PANC-1 cells were transfected with either siScramble or siNPM1 (Supplementary Fig. S6G) and, following reseeding, exposed to RPMI medium, PANC-1 CM, or HMGA1 KO PANC-1 CM. NPM1 knockdown significantly impaired the proliferation only when cells are cultured with PANC-1 CM, while no significant differences were observed in presence of HMGA1 KO PANC-1 CM or RPMI medium (Fig. 5H). These findings further support the functional involvement of NPM1 in this context and underscore its critical role in mediating extracellular HMGA1-dependent cell growth.

Overall, our findings unveil a novel mechanism in which chemotherapeutic treatments activate p53 by inducing Ser15 phosphorylation. Interestingly, wt and mutp53 play opposing roles: while wtp53 inhibits CK2 activity, mutp53 instead enhances it. This shift drives the dysregulation that fuels HMGA1 hypersecretion. Once in the extracellular space, HMGA1 initiates autocrine or paracrine signaling leading to the hyperphosphorylation of NPM1, thereby promoting tumor proliferation.

## Discussion

Our research uncovers a novel and critical mechanism by which mutp53 drives tumor proliferation in PDAC through the hypersecretion of HMGA1. Recognized as one of the deadliest cancers, PDAC often carries *TP53* mutations, which, through their gain-of-function effects, are known to impact the secretion of signaling molecules, driving processes such as tumorigenesis, immune evasion, drug resistance, and metastasis. [12, 17]. Our findings advance current understanding of this complex scenario by identifying HMGA1 as a key secreted protein in mutp53-driven tumors. This discovery may open new therapeutic possibilities to potentially counteract tumor proliferation and invasiveness in *TP53*-mutant PDAC patients. Indeed, identifying cancer-supportive pathways is crucial for enhancing the effectiveness of PDAC treatments and improving patient outcomes. To define and validate the clinical relevance of HMGA1 in PDAC patients, we extensively leveraged multiple datasets. Our findings reveal that HMGA1 expression is significantly higher in PDAC tissues, especially in patients with advanced stages of pancreatic cancer, than in healthy ones. This aligns with multiple studies showing elevated HMGA1 levels in various cancers, including esophageal, colorectal, breast, and gastric cancers, where high HMGA1 expression correlates with advanced disease and poorer prognosis, underscoring its role in tumor progression and metastasis [50–57].

While extensive research has explored the intracellular roles of HMGA1, particularly focusing on its function as a transcription factor, very little was known about its effects once the protein is secreted in the extracellular space. Indeed, prior to our work, only one study has demonstrated that HMGA1 can be actively secreted by triple-negative breast cancer cells, where it promotes metastasis through receptor for advanced glycation end products (RAGE)-mediated activation of phosphorylated extracellular signal-regulated kinase (pERK) signaling, enhancing cancer cell migration and invasion [36].

Our study reveals a novel facet of HMGA1 secretion, showing that it is hypersecreted specifically by human PDAC cell lines carrying GOF *TP53* missense mutations. This finding highlights HMGA1 as a key driver of aggressive behavior observed in *TP53*-mutant tumors, suggesting its critical role in the pathogenesis of these cancers. Furthermore, we demonstrated that chemotherapeutic treatments selectively amplify HMGA1 secretion in these *TP53*^*mut*^ cell lines, underscoring a pivotal role of mutp53 in promoting HMGA1 secretion. Remarkably, restoring wild-type p53 function with Eprenetapopt (APR-246) led to a significant reduction in HMGA1 secretion, even in the presence of chemotherapy. This represents novel mechanism of functional p53, whereby the abrogation of HMGA1 release results in tumor suppression. This finding highlights the potential of molecules that reactivate mutant p53 as targeted, tumor-suppressive strategies for treating p53-mutant cancers, with the added benefit of enhancing treatment efficacy by inhibiting HMGA1 secretion.

Building on previous studies identifying CK2-mediated phosphorylation as essential for HMGA1 secretion [36, 41], we validated the crucial involvement of CK2 in this pathway. Further investigation into the link between mutant p53 and CK2 activity will be essential for understanding the oncogenic properties of *TP53*-mutant tumors. Mutp53 actively enhances CK2 activity, in contrast to the inhibitory effect of wild-type p53 [42]. This dysregulation of CK2 may contribute to the aggressiveness of these tumors, especially considering the findings of this study that demonstrated that chemotherapy further amplifies CK2 activity. Understanding this relationship positions CK2 as a promising therapeutic target for *TP53*-mutant cancers, where its overactivation drives HMGA1 hypersecretion, fueling tumor progression and resistance. Therapeutic strategies targeting CK2, such as the CK2 inhibitor CX-4945, are being explored across several cancers, including leukemia, breast, and prostate cancers, with preclinical data and ongoing clinical trials suggesting that CK2 inhibition could enhance chemotherapy efficacy and reverse resistance mechanisms [58, 59].

Extracellular HMGA1 plays a pivotal role in triggering both autocrine and paracrine signaling, which leads to the phosphorylation of Nucleophosmin1 (NPM1). This phosphorylation event contributes to tumor cell proliferation, thereby sustaining and amplifying the aggressive features of the tumor. While this mechanism is consistent with previous studies showing that activated NPM1 supports tumor cell proliferation, migration, and invasion, primarily via the EGFR/MAPK signaling pathway [60, 61], the precise molecular underpinnings remain to be fully elucidated. By driving these key oncogenic processes, HMGA1 and NPM1 may contribute to the enhanced malignancy and therapeutic resistance observed in PDAC.

Overall, our findings reveal that anticancer treatments enhance CK2-mediated HMGA1 secretion in *TP53*-mutant PDAC cells, which, through autocrine signaling, drives NPM1 hyperphosphorylation and fuels tumor cell proliferation. These insights suggest that targeting any step of this process—whether it be CK2 activity, HMGA1 secretion, the NPM1 phosphorylation cascade—could be a powerful strategy to disrupt this oncogenic pathway and ultimately improving patient outcomes.

## Materials and methods

### Cell culture

The PDAC human cell lines AsPC-1 (p53-null) and PANC-1 (*TP53*^*R273H*^*)* were purchased from the American Type Culture Collection (ATCC). SUIT-2 (*TP53*^*R273H*^*)* and CFPAC (*TP53*^*C242R*^*)* were kindly provided by Prof. S. Ugel (University of Verona, Italy), while PaCa3 (*TP53*^*wt*^*)* and Hs776t (*TP53*^*wt*^*)* by Prof. A. Scarpa (University of Verona, Italy).

For routine propagation, unless otherwise indicated, all cell lines were cultured in RPMI 1640 (Gibco, 52400025) supplemented with 10% FBS (Gibco, A5256701) and 50 μg/mL Gentamicin (Gibco, 15750037) at 37 °C and 5% CO_2_. PBS (Gibco, 14190094) was used for cell washing steps unless otherwise indicated. Cells were regularly tested for Mycoplasma. For treatments, the following drugs and inhibitors were used: Gemcitabine (GEM; Cayman Chemical, 9003096), Oxaliplatin (OXA; Accord Healthcare Italia SRL, Guj/Drugs/G/28/1336), Irinotecan (IRI; Fresenius Kabi, HP/156/06), Fluorouracil (5-FU; Teva Pharmaceuticals, 026542050), Eprenetapopt (APR-246; Selleckchem, S7724), 4,5,6,7-tetrabromobenzotriazole (TBB; Selleckchem, S5265), NSC348884 (Selleckchem, S8149).

### Plasmids extraction and purification

Plasmids pcDNA3.1^+^/C-(K)-DYK and TP53_OHu20059D_R273H_pcDNA3.1^+^/C-(K)-DYK (GenScript) were transformed to DH5α competent cells and amplified in Luria-Bertani (LB) medium (Millipore, 51208) containing the specific antibiotic (Ampicillin sodium salt, Sigma-Aldrich, A9518) overnight at 37 °C using a shaker at 225 rpm. Then, the bacteria were harvested, and the plasmids were extracted and purified using a Qiagen plasmid extraction and purification kit (Qiagen, 12162) according to manufacturer’s instruction.

### Transient transfection

4 × 105 AsPC-1 cells, 3.5 × 105 PANC-1 cells and 3 × 105 HMGA1 KO PANC-1 cells per well were seeded in 6-well plate for 24 h in the growth medium. Transient transfection was performed using Lipofectamine 3000 Reagent (Invitrogen, L30000015) according to the manufacturer’s instructions. For the transfection, Opti-MEM Reduced Serum Medium (Gibco, 31985-062) was used and either plasmid DNAs or siRNAs were added at a concentration of 5 µg/mL or 50 nM, respectively. AsPC-1 cells were transfected for the transient overexpression of mutant *TP53* with *TP53*^*R273H*^ plasmid DNA or MOCK as empty vector control, while PANC-1 cells were transfected for the transient knockdown of the same gene with si*TP53* (Qiagen, SI02655170) or the siScramble as a control siRNA (Qiagen, 102728); HMGA1 KO PANC-1 cells were transfected for the transient knockdown of the *NPM1* gene with si*NPM1* (Qiagen, SI02654967) or the siScramble as a control siRNA (Qiagen, SI02654967).

### Extracellular protein extraction from the conditioned medium (CM)

The medium was replaced with RPMI 1640 without FBS 22 h before the collection. To remove the cells debris, the conditioned medium was centrifuged at 4 °C for 10 min at 1000 × *g* and then 4 times the volume of the sample supernatant of ice-cold acetone was added and placed overnight at −20 °C. Next, the precipitated extracellular proteins were centrifuged at 4 °C for 20 min at 17,000 × g and the pellet was resuspended either in ammonium bicarbonate (Thermo Scientific, 393212500) or RIPA buffer (150 mM NaCl, 50 mM Tris-HCl pH 8, 1% IGEPAL, 0.50% NaDoc, 0.1% SDS) with 1X Halt protease and phosphatase inhibitor cocktail (Thermo Scientific, 1861284) according to the downstream analysis.

### Mass spectrometry-based proteomics

#### Proteomics sample preparation

For in vitro extracellular (conditioned medium) proteomics profiling, PDAC cells were seeded in triplicates in a 6-well plate. After overnight incubation, the cells were transiently transfected and then cultured for further 24 h. Thereafter, the culture medium was aspirated and, after two washes with PBS, replaced with FBS-free medium for further 22 h.

The secreted proteins were extracted as described above and, after the final centrifugation step, the pellets were resuspended in 100 mM ammonium bicarbonate (Thermo Scientific, 393212500) with 1X Halt protease and phosphatase inhibitor cocktail (Thermo Scientific, 1861284). Subsequently, the proteins were quantified by BCA assay (Thermo Scientific, 23227) and the samples diluted to a final concentration of 100 µg of proteins in a final volume of 25 µL of 100 mM ammonium bicarbonate. A comparative analysis was performed between TP53^R273H^ and MOCK AsPC-1 cells [20], siTP53^wt^ and siScramble PaCa3 [62], as previously described, and between siTP53^R273H^ and siScramble PANC-1 cells. Briefly, for each experimental condition, the secreted proteins were reduced with 2.5 µl dithiothreitol (DTT; 200 mM; Sigma-Aldrich, D5545) at 90 °C for 20 min and then alkylated with 10 µL of iodoacetamide (IAM, 200 mM; Sigma-Aldrich, I6709) for 1 h in the dark at room temperature. Thereafter, 2.5 µL of 200 mM DTT were added to destroy the excess of IAM and, after diluting the samples with 300 µL of water and 100 µL of 200 mM ammonium bicarbonate, they were digested with 5 µg of sequencing grade trypsin (Promega, V5111) overnight at 37 °C. The digestion reaction was stopped by adding 2 µL of neat formic acid and the samples were dried with SpeedVac Vacuum Concentrator (Thermo Scientific, model: DNA120-230).

#### LC-MS/MS proteomic analysis

Samples were run on a micro-LC Eksigent Technologies (Sciex) interfaced to a 5600+ TripleTOF mass spectrometer system (Sciex) equipped with a DuoSpray Ion Source and a Calibrant Delivery System (CDS). A Halo Fused C18 (Sciex) was used for the separation. Solven A was 0.1% *v*/*v* formic acid in water. Solvent B was 0.1% *v*/*v* formic acid in acetonitrile. The elution was performed in 30 min at a flow of 15.0 μL/min using increasing concentration (2–40%) of Solvent B to the Solvent A. The injection volume was 4.0 μL and the oven temperature was kept at 40 °C. For the identification, the samples were subjected to data dependent analysis (DDA): the MS operated with a mass range of 100–1500 Da (TOF scan with an accumulation time of 0.25 s), followed by a MS/MS product ion scan from 200 to 1250 Da (accumulation time of 0.5 s) with the abundance threshold set at 30 cps. Ion source parameters in electrospray positive mode: curtain gas (N_2_) at 25 psig, nebulizer gas GAS1 at 25 psig, GAS2 at 20 psig, ionspray floating voltage (ISFV) at 5000 V, source temperature at 450 °C, and declustering potential at 25 V. For the quantification, the samples were subjected to cyclic data independent analysis (DIA) of the mass spectra using a 25-Da window: a 50-ms survey scan (TOF-MS) was performed and subsequent MS/MS experiments were performed in a cyclic manner using an accumulation time of 40 ms per 25-Da swath (36 swaths in total) for a total cycle time of 1.5408 s on all precursors. The MS data were acquired with Analyst TF 1.7 (Ab Sciex). Two DDA and three DIA acquisitions were performed. The DDA files were processed with Protein Pilot software v. 4.2 (Ab Sciex) and Mascot v. 2.4 (Matrix Science Inc). The peptide mass tolerance was 0.08 Da and the MS/MS tolerance was 10 ppm. Monoisotopic mass and peptide charges at 2+, 3+, and 4+ were set for the search. The UniProt Swiss-Prot reviewed database containing human proteins (version 2015.07.07, containing 42131 sequence entries) was used and a target-decoy database search was performed. False Discovery Rate was fixed at 1%. Label-free quantification was performed using Skyline 3.1 (http://proteome.gs.washington.edu/software/skyline, accessed on 10 February 2022).

MSstats (v.2.0) was used for the statistical analysis of proteins and peptides quantitative differences between samples.

#### HMGA1 CRISPR–Cas9 knockout

The human *HMGA1* gene was knockout using the human gene knockout kit via CRISPR, non-homology mediated (Origene, KN401458). For transfection, the human PANC-1 cells were seeded at 4 × 105 cells per well in a 6-well plate a day earlier. The cells were transfected with 1 μg of plasmid using Lipofectamine 3000 Reagent (Invitrogen, L30000015) according to manufacturer’s instruction. After 24 h, the selection of successfully transfected cells was commenced by culturing the cells with 1 µg mL^−1^ puromycin (InvivoGen) in RPMI. The puromycin-containing medium was replaced every 2 days until selection was complete, as indicated by the death and detachment of all non-transfected cells. Thereafter, the successfully transfected cell lines were expanded and clonally selected after serial dilution.

#### Cell invasion assay

Transwell invasion assays (n = 4) were performed using 24-well inserts coated with Matrigel (Corning, Cat. 354230, Lot 8145001; concentration: 8.7 mg/mL). Matrigel was diluted in cold serum-free RPMI to a final concentration of 3 mg/mL, and 100 µL of the diluted solution was added to each insert and allowed to solidify at 37 °C for 4 h. PANC-1 and HMGA1 KO PANC-1 cells were seeded in the upper chamber at a density of 25,000 cells/well in 500 µL of RPMI supplemented with 2% FBS. As a chemoattractant, 750 µL of RPMI containing 20% FBS was added to the lower chamber. After 24 h of incubation at 37 °C, non-invading cells and Matrigel were carefully removed from the upper surface of the inserts using a cotton swab. Invading cells on the lower surface were fixed and stained with a crystal violet solution (0.75% crystal violet (Sigma-Aldrich, C3886), 0.25% NaCl, 1.7% formaldehyde, 50% ethanol) for 10 min at room temperature. The inserts were then washed thoroughly with water and left to dry overnight. Images of the stained membranes were acquired by using an optical microscope (APX100, Olympus, Evident Scientific, Hamburg, Germany) equipped with 4× objective to acquire the entire well and 10× objective to obtain a magnification image. The crystal violet dye was subsequently solubilized using PBS containing 1% SDS (Sigma-Aldrich, 05030) for 1 h. Absorbance was measured at 595 nm using an Infinite M Nano+ Microplate Reader (Tecan, 30190087).

#### Cell proliferation assay

HMGA1 KO PANC-1 cells were seeded in 48-well plates at a density of 20,000 cells per well in 400 µL of growth medium. Next day, the medium was aspirated and the treatment medium introduced (200 µL of PANC-1 CM or HMGA1 KO PANC-1 CM + 200 µL of fresh growth medium) and after 48 h relative proliferation was determined with Crystal violet staining. Briefly, the medium was aspirated and about 400 µL of crystal violet (0.75% Crystal violet (Sigma-Aldrich, C3886), 0.25% NaCl, 1.7% formaldehyde, 50% ethanol) were added. After the incubation period (10 min), the staining was removed and the plate washed in water. After an overnight to let the plate dry, 400 µL of Solubilization buffer (PBS + 1% SDS (Sigma-Aldrich,05030)) were added to each well and incubated for 1 h before measuring absorbance at 595 nm using an Infinite M Nano+ Microplate Reader (Tecan, 30190087).

HMGA1 KO PANC-1 cells were seeded in 96-well plates at a density of 7000 cells per well in 200 µL of growth medium. After 24 h the medium was replaced with fresh growth medium containing varying concentrations of rHMGA1 (0, 0.1, 0.2, 0.4, 0.8, 1 µg/mL). Following 72 h of incubation, relative cell proliferation was assessed using Crystal violet staining as described above (for a 96-well plate, 100 µL of crystal violet and then 100 µL of Solubilization buffer were used).

AsPC-1 cells were seeded in 96-well plates at a density of 10,000 cells per well in 200 µL of growth medium. The following day, the medium was replaced with R273H CM or MOCK CM with the addition of anti-HMGA1 antibody (0.226 µg/µL, 1:100 in growth medium) or the IgG Isotype Control (Cell signaling, 5742). At the end of the experiment duration (48 h), relative proliferation was determined with Crystal violet staining, as described before.

HMGA1 KO PANC-1 cells were seeded in 96-well plates at a density of 7000 cells per well in 200 µL of growth medium. After aspirating the medium, the following treatment conditions media were introduced: PANC-1 CM, HMGA1 KO PANC-1 CM and PANC-1 CM + 0.2 µM of NSC 348884. After 24 h, relative proliferation was determined with Crystal violet staining.

3.0 × 10^5^ HMGA1 KO PANC-1 cells were seeded in 6-well plates. The following day, cells were transfected with siScramble or siNPM1 (as described in the “Transient Transfection” section). 24 h post-transfection, cells were reseeded in sextuplicate into 96-well plates at a density of 7000 cells per well in 200 µL of growth medium. The next day, the medium was aspirated and replaced with one of the following treatment conditions: RPMI, PANC-1 CM, or HMGA1 KO PANC-1 CM. After 24 h incubation, relative proliferation was assessed by Crystal Violet staining.

#### Cell viability by MTT

Cells were seeded in 96-well plates at 7500 cells per well in growth medium and allowed to attach overnight. Following the 24h-treatment period with different drugs and inhibitors at indicated concentrations, MTT solution (Invitrogen, M6494, at final concentration 5.5 mg/mL in PBS) was added for 3 h to the cells. The medium was then replaced with 25 µL of fresh medium and 50 µL of DMSO (Sigma-Aldrich, D2650) to each well to lyse the cells and after 40 min incubation at 37 °C, the absorbance of the formazan product released was measured at 540 nm using an Infinite M Nano+ Microplate Reader (Tecan, 30190087).

#### Cell viability by flow cytometry

To assess the cell viability the eBioscience Annexin V Apoptosis Detection Kit APC (Invitrogen, 88800774) was used in different experimental settings. Cells were seeded in triplicates in a 24-well plate at 7 × 10^4^ cells per well in growth medium. After overnight incubation, the cells were washed two times with PBS and the medium was replaced with fresh RPMI with and without FBS for 22 h or the cells were treated with chemotherapeutic drugs at indicated concentrations for 24 h. Flow-cytometric analysis was done on a BD LSRFortessa (BD Bioscience) using BD FACS Diva software, and data analysis was performed using FlowJo v10.8.1 software.

### Bioinformatic analysis

#### Gene analysis in single-cell RNA seq data

The determination of the *TPM1*, *CLSTN1*, *CYP2R1*, *HMGA1 CSNK2A1, CDK1, CDK2, HIPK2* and *NPM1* gene expression at single-cell level was done using single-cell RNA sequencing data from healthy donor samples (*n* = 6) [22] integrated with previously published dataset [23] of tumor (*n* = 16) and adjacent normal (*n* = 3) samples available in the Pancreatic Tissue Single Cell Atlas (https://pascadimaglianolab.shinyapps.io/SC_Pancreas_Atlas/, accessed in June 2025). Feature matrices of scRNA-seq data are available from the NIH Gene Expression Omnibus database under the accession number GSE229413 and GSE155698.

#### HMGA1 gene analysis in RNA sequencing dataset

*HMGA1* gene expression analysis was performed in tumor (*n* = 179) compared to normal (*n* = 171) samples using GEPIA database (http://gepia.cancer-pku.cn/index.html, accessed in November 2024) [63]. TCGA and GTEx pancreatic adenocarcinoma data were used as RNA sequencing expression data. Log_2_(TPM + 1) was used for log-scale. 1 was used as cut off for log_2_FC and 0.01 for p-value. *HMGA1* mRNA expression analysis was performed in *TP53* wild-type and mutant PDAC patients using the *Pancreatic Adenocarcinoma TCGA PanCancer* data (*n* = 184) [64–73] as patients RNA seq data from cBioportal (https://www.cbioportal.org, accessed in November 2024). Differential *HMGA1* mRNA expression analysis in PDAC patients carrying wild-type or mutant *TP53* was performed also using the *QCMG* data (*n* = 456) [5] from cBioportal (https://www.cbioportal.org, accessed in November 2024).

#### Kaplan-Meier overall survival

The overall survival of PDAC patients (*n* = 536) was analyzed using KM plotter database (https://pancreas.kmplot.com/, accessed in October 2024). The patients were split by trichotomization. Then, the Kaplan-Meier plot was plotted also after stratifying PDAC patient for tumor stage (S2, S3, S4) (*n* = 294). The statistical significance between groups was evaluated using the log-rank test. Hazard ratios (HRs) with 95% confidence intervals were calculated and reported where applicable.

#### PDAC dataset analysis

The human PDAC microarray datasets with accession numbers GSE71729 (*n* = 61 metastatic PDAC tumors vs 145 primary) [74] was obtained from NCBI GEO (https://www.ncbi.nlm.nih.gov/geo/) [75]. GEO2R was used to compare the two groups of samples and identify genes that are differentially expressed across experimental conditions. Default parameters were applied for data processing and analysis, with adjusted p-values calculated using the Benjamini & Hochberg method to control the false discovery rate (FDR).

#### Bioinformatic analysis of HMGA1 post-translational modifications

The analysis of post-translational modification (PTM) sites on HMGA1 was conducted using the PhosphoSitePlus® PTM Database (version 6.8.0) [76]. We specifically investigated the most frequently reported phosphorylation sites by selecting the High Throughput Paper modality. This filter includes only those sites identified exclusively through mass spectrometry-based proteomic discovery approaches.

To ensure robustness and recurrence of the modifications, we applied an additional filter by selecting PTM sites supported by a minimum of five independent references. This criterion allowed us to focus on high-confidence phosphorylation events.

In addition, the database was queried to display putative in vivo kinases predicted to target HMGA1, along with the corresponding phosphorylation sites. The information was retrieved and visualized directly through the PhosphoSitePlus® interface.

#### Reverse transcription with qPCR

PANC-1 cells were seeded in a 6-well plate at a density of 4 × 10^5^ cells per well, allowed to attach overnight and then treated with 1 µM GEM for different times (6 h, 10 h, 24 h, 48 h).

Thereafter, cells were washed with PBS, lysed with 600 µL of Trizol Reagent (Invitrogen, 15596018), and transferred to 1.5 mL tubes. After 5 min at room temperature, 120 µL of chloroform was added, mixed for 15 sec, and incubated for 3 min. Samples were centrifuged at 4 °C for 15 min at 12,000 × *g*, and the aqueous phase was collected. RNA was precipitated with 300 µL of isopropanol and 1.5 µL of GlycoBlue Coprecipitant (Invitrogen, AM9516), then centrifuged at 4 °C for 15 min at 12,000 × *g*. Pellets were washed with 70% ethanol, centrifuged at 7500 × *g* for 5 min, air-dried, and resuspended in RNase-free water.

RNA purity and quantification was assessed using a NanoDrop One (ThermoFisher Scientific, ND-ONE-W). Thereafter, 1 μg of the RNA samples were reverse transcribed to cDNA using the High Capacity cDNA Reverse Transcription Kit (ThermoFisher Scientific, 4368813) according to the manufacturer’s instructions. qPCR was performed on the QuantStudio 3 Real-Time PCR System (ThermoFisher Scientific, A28137) using GoTaq qPCR Master Mix (Promega, A6001). The reactions were run at 10 µL total volume consisting of 5 µL Master Mix, 3 µL nuclease free water, 2 µL of cDNA after diluting 1:20 in water, 0.04 µL of 100 µM forward (F) primer, and 0.04 µL 100 µM reverse (R) primer. *HMGA1* (Forward: CAACTCCAGGAAGGAAACC; reverse: AGGACTCCTGCGAGATGC) and *TP53* (Forward: GGCCCACTTCACCGTACTAA; reverse: GTGGTTTCAAGGCCAGATGT). The comparative cycling threshold (ΔΔ*Ct*) method was used to analyze the qPCR data and *GAPDH* (Forward: ATCAGCAATGCCTCCTGCAC; reverse: TGGTCATGAGTCCTTCCACG) was used as a housekeeping gene.

#### Western blot analysis

Following culture, the FBS-free medium was processed for the extracellular protein extraction as previously described, and the wells washed one time with PBS. The pellet of extracellular proteins was resuspended in 150 µL of RIPA buffer (150 mM NaCl, 50 mM Tris-HCl pH 8, 1% IGEPAL, 0.50% NaDoc, 0.1% SDS) with 1X Halt protease and phosphatase inhibitor cocktail (Thermo Scientific, 1861284) and quantified using the Bradford Protein Assay Kit (Thermo Scientific, 1856209) according to the manufacturer’s instructions. To load 40 µg protein per lane of secretome samples, a second overnight precipitation step at −20 °C with ice-cold acetone was required. Thereafter, the samples were centrifuged at 4 °C for 10 min at 14,000 × *g* and the pellets resuspended in 13 µL of RIPA buffer. Meanwhile, 100–150 μL of RIPA buffer containing phosphatase and protease inhibitors, were transferred to each well to lyse the cells. Lysis and the collection of the lysates were completed on ice. Following a 10-min incubation on ice, lysates were collected into 1.5 mL tubes and centrifuged at 4 °C for 10 min at 15,000 × *g* to extract the sample supernatant. Protein concentration of the proteomes for western blot analysis was measured using Bradford Protein Assay Kit (Thermo Scientific, 1856209) according to the manufacturer’s instructions. The proteome samples were prepared at 25 µg protein. After the addition of Sample Buffer (4X; 0.2 M Tris-HCl pH 6.8, 8% SDS, 40% glycerol, Bromophenol blue) and 2-mercaptoethanol (10X; Thermo Scientific, 35602BID), both the secretomes and the proteomes were boiled for 5 min at 98 °C. Samples were run on a 15% SDS-PAGE gel at 120 V alongside Precision Plus Protein Dual Color Standards (Biorad, 1610374) and transferred to methanol-activated PVDF membranes (Thermo Scientific, 88518) at 75 V for 1 h and 30 min. Membranes were blocked with 5% non-fat dried milk in TBS-T solution (tris-buffered saline with 0.1% Tween-20, Sigma-Aldrich, P1379) for 1 h at room temperature and incubated overnight at 4 °C with primary antibodies in blocking buffer. After three TBS-T washes, membranes were incubated with secondary antibodies (1:2000) for 1 h at room temperature, washed again, and incubated with chemiluminescence reagent (SuperSignal West Pico PLUS Chemiluminescent Substrate, Thermo Scientific, 34580) according to the manufacturer’s instructions. Subsequently, blot images were acquired on a Bio-Rad ChemiDoc™ MP Imaging System (12003154) (Image Lab Touch Software version 3.0.1.14). The following primary antibodies were used in this study and at 1:1000 dilution: anti-HMGA1 (Cell Signaling, 7777S), anti-P53 (Cell Signaling, 2527S), anti-pP53 (Ser15) (Cell Signaling, 9286S), anti-phospho-CK2 substrate (Cell Signaling,8738), anti-cleaved PARP (Cell Signaling, 5625), anti-pNPM1 (T199) (Abclonal, AP0836), anti-NPM1 (Abclonal, A17983), anti-Vinculin (Cell Signaling, 13901S). The following secondary antibodies were used: mouse anti-rabbit IgG-HRP (Santa Cruz, sc-2357), m-IgGκ BP-HRP (Santa Cruz, sc-516102). Vinculin was used as a loading control for the proteome samples, while amido black (a.b.) staining for the secretomes.

### Phosphoproteomics analysis

#### Sample preparation for LC-MS phosphoproteomics analysis

PANC-1 and HMGA1 KO PANC-1 cells were seeded in T75 flasks at a density of 2.5–3.5 × 10^6^ cells, allowed to attach overnight and then treated with 1 µM GEM for 24 h. Next, the medium was changed with fresh medium. After 22 h, PANC-1 CM and HMGA1 KO PANC-1 CM were collected and centrifuged for 5 min at 1000 × *g*. The HMGA1 KO PANC-1 cells, that were previously seeded in quadruplicate in T75 flasks at a density of 4 × 10^6^ cells, were incubated with the PANC-1 CM and the HMGA1 KO PANC-1 CM at 37 °C and 5% CO_2_ for 30 min. Thereafter, the cells were pelleted and each sample lysed with 400 µL of urea lysis buffer (8 M urea, 75 mM NaCl, 50 mM Tris pH 8, 1 mM EDTA, 1X Protease and Phosphatase inhibitor cocktail) 30 min on ice and vortexed every 10 min [77]. Then, the lysates were centrifuged for 10 min at 10,000 × *g* and the protein concentration was quantified using Pierce BCA Protein Assay Kit (Thermo Scientific, 23225) according to the manufacturer’s instructions. Equal amounts of protein (2000 µg) from each sample were reduced with 5 mM dithiothreitol (DTT; Thermo Scientific, R0861) at room temperature for 45 min in the dark and alkylated with 10 mM 2-iodoacetamide (IAM; Thermo Scientific, A14715) at room temperature for 45 min in the dark. Next, the proteins were digested with Pierce Trypsin Protease (1:50 enzyme:protein) (Thermo Scientific, 90059) overnight at 37 °C in Low Binding 1.5 mL tubes (Thermo Scientific, 90410). The samples were acidified with neat formic acid, centrifuged for 5 min at 2000 × *g* to remove the precipitate and dried with SpeedVac Vacuum Concentrator (model: Savant DNA 120 SpeedVac Concentrator, Thermo Scientific). Next, the peptides were desalted using the Pierce™ Peptide Desalting Spin Columns (Themo Scientific, 89852) according to the manufacturer’s instructions. Thereafter, the phosphopeptides were enriched using the High-Select™ TiO_2_ Phosphopetide Enrichment Kit (Thermo Scientific, A32993), subsequently the TiO_2_-flowthrough was further enriched using the High-Select™ Fe-NTA Magnetic Phosphopeptide Enrichment kit (Thermo Scientific, A52283) according to the manufacturer’s instructions. The dried phosphopeptide pellets were resuspended using 0.1% Formic acid (50 µL) (Thermo Scientific, 85170) and quantified using the Pierce Quantitative Fluorimetric Peptide Assay (Thermo Scientific, 23290), according to the manufacturer’s instructions, before the LC-MS analysis.

#### LC-MS phosphoproteomics analysis

LC-ESI-MS/MS analysis was performed using an Ultimate 3000 nanoUPLC system (Thermo Fisher Scientific) coupled to an Orbitrap Fusion Lumos Tribrid mass spectrometer (Thermo Fisher Scientific). For reversed-phase UPLC separation, 1 µg of the phosphopeptide mixture was loaded onto an Easy-Spray PepMap RSLC C18 analytical column (2 µm, 500 × 0.075 mm, Thermo Fisher Scientific) and separated using a gradient from 4% to 50% acetonitrile over 90 min. Ionization was achieved with a nanoESI source in positive ion mode. MS^1^ spectra were acquired in data-dependent acquisition (DDA) mode using the Orbitrap analyzer, with an m/z range of 375–1500, a resolution of 120,000 (at 200 m/z), standard automated gain control (AGC), and a maximum injection time of 50 ms. MS^2^ spectra were also obtained using the Orbitrap analyzer, operating at a resolution of 50,000 (at 200 m/z). Precursors were selected based on their intensity from all signals with a charge state of 2+ to 5+, isolated in a 2.0 Da window, and fragmented using higher-energy collisional dissociation (HCD) with a dynamic exclusion of 60 s. Each biological replicate was analyzed in quadruplicate for reliable data acquisition.

LC-ESI-MS/MS data analysis was performed using Proteome Discoverer (v2.5), employing the Sequest HT search engine for protein identification. Carbamidomethyl (C) was set as a fixed modification, while oxidation (M) and acetylation (protein N terminus) as variable modifications. To identify phosphorylation sites, we applied the ptmRS node, specifically targeting serine (S), threonine (T), and tyrosine (Y) residues. The Uniprot database was used for protein identification, with trypsin specified as the protease and a maximum allowance of two missed cleavages. We used the “match between runs” option to transfer peptide identifications across samples within 2 min of the aligned retention times. Peptide identifications confidence was calculated using the Percolator algorithm with decoy database searching. Identifications were filtered by FDR validation based on the q value, with a strict threshold of 0.01 and a relaxed threshold of 0.05. Label-free quantification of identified proteins was conducted using unique peptides, requiring a minimum ratio count of two, and calculated using raw spectral protein intensities. For each condition, raw intensities were log-transformed, normalized to the calculated average, and used for downstream analyses. Student’s t-test was performed on the normalized protein intensities, and proteins showing p < 0.05 and a fold change >1.5 were considered significantly altered in abundance between the samples.

#### Protein-protein interactions (PPIs) network analysis

The 101 significantly dysregulated proteins identified by the phosphoproteomics analysis were uploaded into STRING database (Version:12.0) (https://string-db.org) to construct the PPIs network. The analysis was performed limited to “*Homo sapiens*” as a species, including experiments, databases and co-expression as active interaction sources and setting a medium confidence (0.400) as confidence score.

The STRING results were then analyzed using CytoHubba Cytoscape (Version**:** 3.10.2) plugin to identify the hubs between the interacting proteins. The ranking mode selected was the betweenness.

#### Gene co-expression analysis in single-cell RNA seq data

The level of *HMGA1* and *NPM1* genes co-expression at single-cell level was done using single-cell RNA sequencing data from tumor donor samples (*n* = 16) available in the Pancreatic Tissue Single Cell Atlas (https://pascadimaglianolab.shinyapps.io/SC_Pancreas_Atlas/, accessed in October 2024).

#### Gene co-expression analysis in cBioportal

To investigate the transcriptional association between *HMGA1* and *NPM1*, we performed a co-expression analysis using two independent Pancreatic Adenocarcinoma dataset: the Pancreatic Adenocarcinoma (TCGA, GDC) dataset (mRNA expression data from n = 179 samples) and the Pancreatic Ductal Adenocarcinoma (CPTAC, Cell 2021) dataset (mRNA expression (log2 RSEM uq) from n = 140 samples), accessed via the cBioPortal for Cancer Genomics (https://www.cbioportal.org/, accessed in June 2025). Spearman’s rank correlation coefficient (ρ) and Pearson’s correlation coefficient (r) were used to assess the strength of association between gene expression levels. Statistical significance was calculated using two-sided t-test.

#### Mice

NOG mice (NOD.Cg-Prkdc^scid^Il2rg^tm1Sug^/JicTac) were purchased from Taconic (Hudson, New York, USA). Ten-weeks-old male mice were separated in two groups (*n* = 6, each group) each corresponding to a specific tumor cell line. 1 × 10^6^ PANC-1 or HMGA1 KO PANC-1 cells were inoculated into the left flank of mice subcutaneously. Tumor volume was weekly determined by digital caliper from 11 days after tumor challenging to the endpoint of the experiment (day 60).

Blinding was applied during the in vivo experiment. Mice were randomly assigned numerical codes by an operator and housed in mixed cages to avoid group identification. Tumor volume measurements were then performed by a second operator who was blinded to group allocation, in order to minimize assessment bias.

Randomization was not applicable, as group allocation was determined by the cell line used for tumor inoculation. No animals were excluded from the analysis; however, mice would have been excluded if they had failed to develop a palpable tumor reaching at least 100 mm³ by the end of the study. All mice were maintained under specific pathogen-free conditions in the animal facility of the University of Verona. Food and water were provided ad libitum. Animal experiments were performed according to national protocol number C46F4.26 approved by the Ministerial Decree Number 993/2020-PR of July 24, 2020 and protocol number C46F4.30 approved by the Ministerial Decree Number 227/2023-PR of March 14, 2023 [PI: Stefano Ugel] and Europeans laws and regulations. All animal experiments were approved by by Animal Welfare Organization (OPBA) (https://www.univr.it/it/ateneo/organismo-preposto-al-benessere-degli-animali-opba) and conducted according to the guidelines of Federation of European Laboratory Animal Science Association (FELASA). All animal experiments were in accordance with the Amsterdam Protocol on animal protection and welfare: mice were monitored daily and euthanized when displaying excessive discomfort.

#### Recombinant human HMGA1

To produce the recombinant human HMGA1 protein (rHMGA1) the pET-6xHis/TEV/hHMGA1[NM_145899.3](co)*(Del_ATG) expression vector from VectorBuilder (catalog no. VB240207-1386fxa) was used. Protein expression was carried out in Rosetta2 (DE3) cells, initially grown overnight in 10 mL LB medium containing ampicillin (Amp). 10 mL per 1 L of TB with Amp were inoculated and cultured at 37 °C until reaching an OD600 of 1.0. The temperature was reduced to 20 °C for 1 h prior to induction. Protein expression was induced with 0.4 mM IPTG, and incubation was continued at 20 °C overnight. Finally, cells were harvested by centrifugation at 5000 rpm for 15 min. To purify rHMGA1 protein, the cell pellet (from a 1 L culture) was resuspended in 40 mL of lysis buffer (PBS with 40 µL Leupeptin/Aprotinin). Then, the cells were lysed via ultrasonication for a total of 3 min in cycles of 6 × 30 s, with 30-s pauses in between. After that, to obtain a clear supernatant, the lysate was centrifuged at 18,000 rpm in an SS34 rotor (Thermo Scientific) for 45 min. Before adding the cleared lysate, 5 mL of Ni-NTA (Qiagen Cat# 30450) resin were pre-equilibrate with lysis buffer in a column, then it was rotated at 4 °C for 1 h. The resin was subsequently washed five times with 15 mL of Ni Wash Buffer 1 (PBS with 10 mM imidazole), and the protein was eluted using 25 mL of Ni Elution Buffer 1 (PBS with 300 mM imidazole). For TEV protease cleavage and further purification, 500 µL of TEV protease (1 mg/mL) per 50 mg of eluted protein were added and the eluted protein was, then, dialyzed overnight at 4 °C in a YM-10 dialysis tube with 1 L of dialysis buffer (PBS with 1 mM DTT). Following dialysis, the Ni resin was pre-equilibrated with Ni Wash Buffer 2 (PBS), then the cleaved protein was loaded onto the Ni resin and incubated at 4 °C for 1 h. The flow-through, which contains hHMGA1, was collected and combined with the elution obtained after washing the resin with 5 mL of Ni Wash Buffer 2 (PBS). The flow-through was concentrated in a Amicon YM-10 concentrator (Sigma Cat # UFC901096), then applied to an S75 column. The fractions were pooled and analyzed by SDS-PAGE. The pooled protein exhibited an A(260/280) ratio of 1.12, suggesting DNA contamination. To address this, the protein was loaded onto a HiTrap Heparin column in PBS and it came in the flow-through without sticking to the column. Finally, the protein was concentrated to 1 mg/mL in PBS for further use.

### Statistical analysis and software

Results are expressed as mean ± s.d., except for the in vivo experiment where data are presented as mean ± s.e.m. Significance between 2 groups was tested using a 2 tailed unpaired t test, assuming similar variances between groups. Significance among multiple groups was tested using a one-way ANOVA, which also assumes homogeneity of variances. GraphPad Prism 10.3.1 was used for the statistical analysis. Statistical significance is described in the figure legends as: ∗ p < 0.05, ∗∗ p < 0.01, ∗∗∗ p < 0.001, ∗∗∗∗ p < 0.0001, unless otherwise specified. If data did not meet the assumptions of the originally intended parametric test (e.g., normal distribution or homogeneity of variance), an alternative non-parametric test was applied. In such cases, the specific statistical test used is clearly indicated in the corresponding “Materials and Methods” section or figure legend.

The Venn diagram was made using DeepVenn (https://www.deepvenn.com/) Hulsen, T. (2022). DeepVenn--a web application for the creation of area-proportional Venn diagrams using the deep learning framework. Image Lab Software 6.1 was used to analyze the blot images. Flow cytometer data analysis was performed using FlowJo software v10.8.1. For the bioinformatic analyses were used: Pancreatic Tissue Single Cell Atlas (https://pascadimaglianolab.shinyapps.io/SC_Pancreas_Atlas/), GEPIA database (http://gepia.cancer-pku.cn/index.html), cBioportal for Cancer Genomics (https://www.cbioportal.org/), KM plotter database (https://pancreas.kmplot.com/), NCBI GEO (https://www.ncbi.nlm.nih.gov/geo/), PhosphoSitePlus® PTM Database (version 6.8.0) (https://www.phosphosite.org/homeAction.action), STRING database (Version:12.0) (https://string-db.org), CytoHubba Cytoscape (Version**:** 3.10.2) plugin. Figures were created using Biorender.com.

## Supplementary information

Supplementary materials

Supplementary Table 1

Supplementary Table 2

Full and uncropped western blots

## Data Availability

All data generated or analyzed during this study are included in this published article [and its supplementary information files]. Unprocessed original Western blot images are included in the supplementary material. The mass spectrometry proteomics data have been deposited to the ProteomeXchange Consortium via the PRIDE partner repository with the dataset identifier PXD062127. Any supplementary information can be made available from the lead contact upon a reasonable request.
