# Supplementary material for: Chemotherapy enhances HMGA1 secretion through the mutant p53-CK2 axis in pancreatic ductal adenocarcinoma cells
Source: Cell Death Dis. 2025 Oct 27;16(1):766. doi: 10.1038/s41419-025-08082-1 (PMC12559235; doi:10.1038/s41419-025-08082-1)
Supplement: Supplementary file 1 — Supplementary materials [file 41419_2025_8082_MOESM1_ESM.pdf]

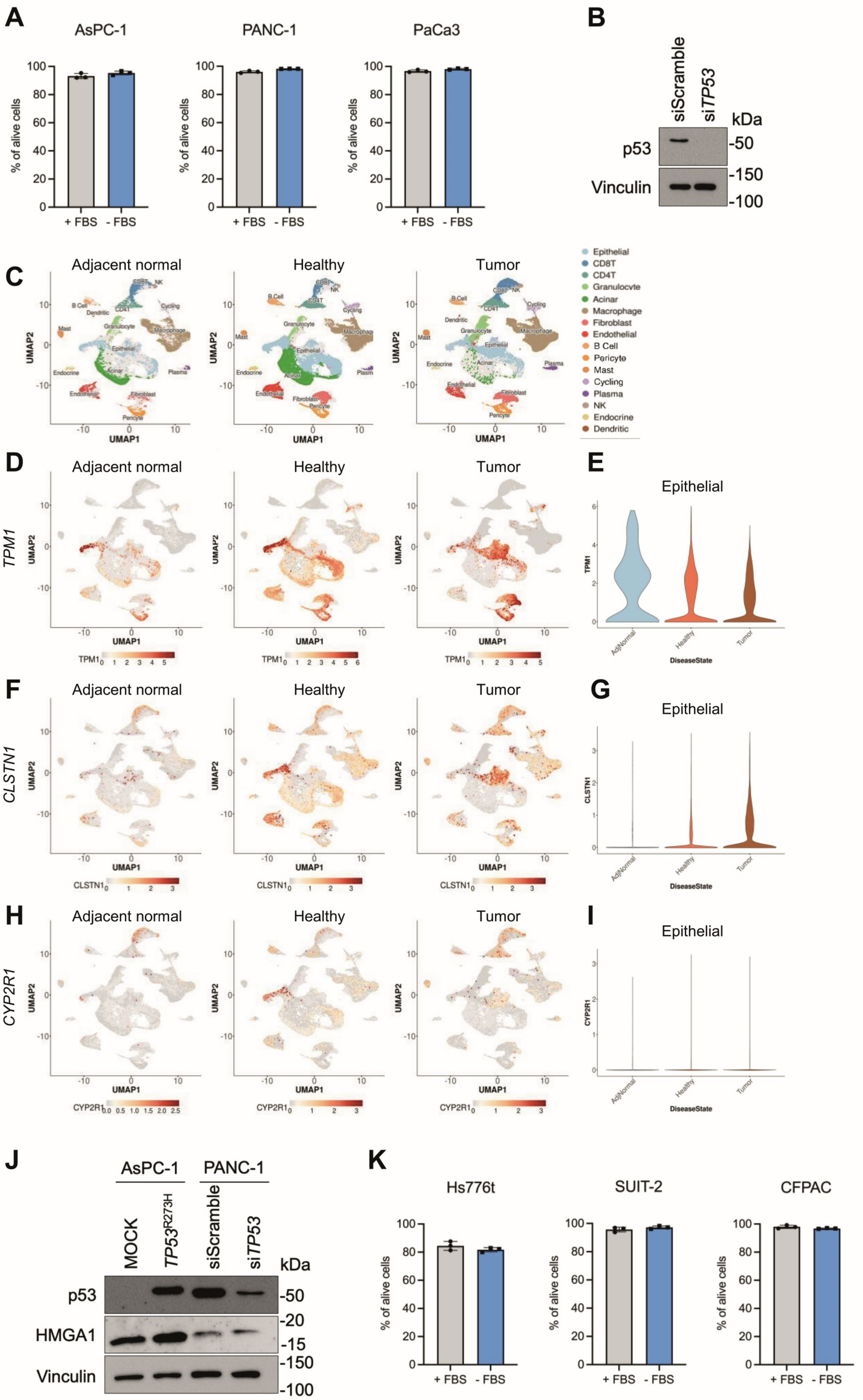

A
AsPC-1
PANC-1
PaCa3
% of alive cells
+ FBS
- FBS
B
siScramble
siTP53
kDa
p53
Vinculin
-50
-150
-100
C
Adjacent normal
Healthy
Tumor
UMAP1
UMAP2
Epithelial
CD8T
CD4T
Granulocyte
Acinar
Macrophage
Fibroblast
Endothelial
B Cell
Pericyte
Mast
Cycling
Plasma
NK
Endocrine
Dendritic
D
TPM1
E
Epithelial
DiseaseState
AdjNormal
Healthy
Tumor
F
CLSTN1
G
H
CYP2R1
I
J
AsPC-1
PANC-1
MOCK
TP53R273H
siScramble
siTP53
p53
HMGA1
Vinculin
-50
-20
-15
-150
-100
K
Hs776t
SUIT-2
CFPAC

Supplementary Figure 1: Gene expression analysis of mutp53-dependent secreted proteins in PDAC datasets.

(A) Bar charts of AsPC-1, PANC-1 and PaCa3 cells viability (PI-/Ann V-) after 22 h treatment with or without 10% FBS. **(B)** Immunoblot of p53 protein in PANC-1 cells after *TP53* silencing. Vinculin was used as a loading control. **(C)** UMAP visualization of pancreatic microenvironment cell types by disease state: Adjacent Normal (*n* = 3), Healthy (*n* = 6), and Tumor (*n* = 16). Data source: Pancreatic Tissue Single Cell Atlas. **(D, F, H)** UMAP visualizations showing *TPM1*, *CLSTN1* and *CYP2R1* expression, respectively, across major cell populations subset by disease state (Adjacent Normal, Healthy, Tumor). Data source: Pancreatic Tissue Single Cell Atlas. **(E, G, I)** Violin plots comparing *TPM1*, *CLSTN1* and *CYP2R1* gene expression, respectively, in epithelial cells from Adjacent Normal (n = 892), Healthy (n = 14,380), and Tumor (n = 9,484) samples. Data source: Pancreatic Tissue Single Cell Atlas. **(J)** Immunoblot of p53 and HMGA1 proteins in AsPC-1 and PANC-1 cells after being transiently transfected to overexpress or silence *TP53*, respectively. **(K)** Bar charts of Hs776t, SUIT-2 and CFPAC cells viability (PI-/Ann V) after 22 h treatment with or without 10% FBS.

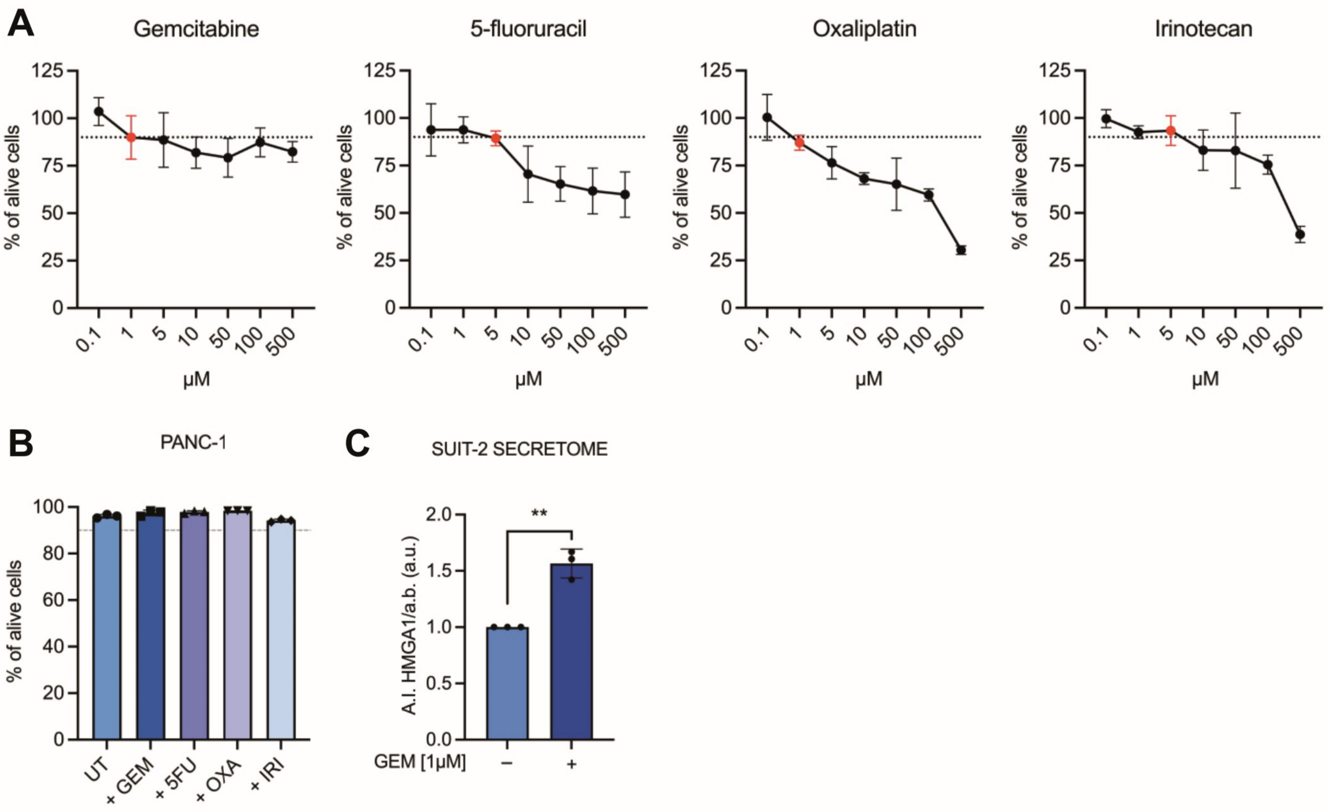

A
Gemcitabine
5-fluoruracil
Oxaliplatin
Irinotecan
% of alive cells
0.1
1
5
10
50
100
500
μM
B
PANC-1
% of alive cells
UT
+ GEM
+ 5FU
+ OXA
+ IRI
C
SUIT-2 SECRETOME
A.I. HMGA1/a.b. (a.u.)
**
GEM [1μM]
–
+

Supplementary Figure 2: Chemotherapeutic drugs treatments promote mutp53-driven HMGA1 hypersecretion.

(A) Identification of the highest dose of chemotherapy able to preserve the human PANC-1 cells viability ≥ 90%. **(B)** Cells viability of PANC-1 cells after 24 h treatment with 1 μM GEM, 5μM 5-FU, 1μM OXA and 5μM IRI. Horizontal dashed line indicates 90% of alive cells (PI-/Ann V-) threshold. **(C)** Bar charts depict A.I. (a.u.) of HMGA1 secreted by SUIT-2 cells with or without 1 μM GEM treatment versus a.b. analyzed using Image Lab Software (Bio-Rad, version 6.1.0 build 7). Data plotted are mean of three independent experiments +/- SD. (Unpaired t-test). ** $p<0.01$.

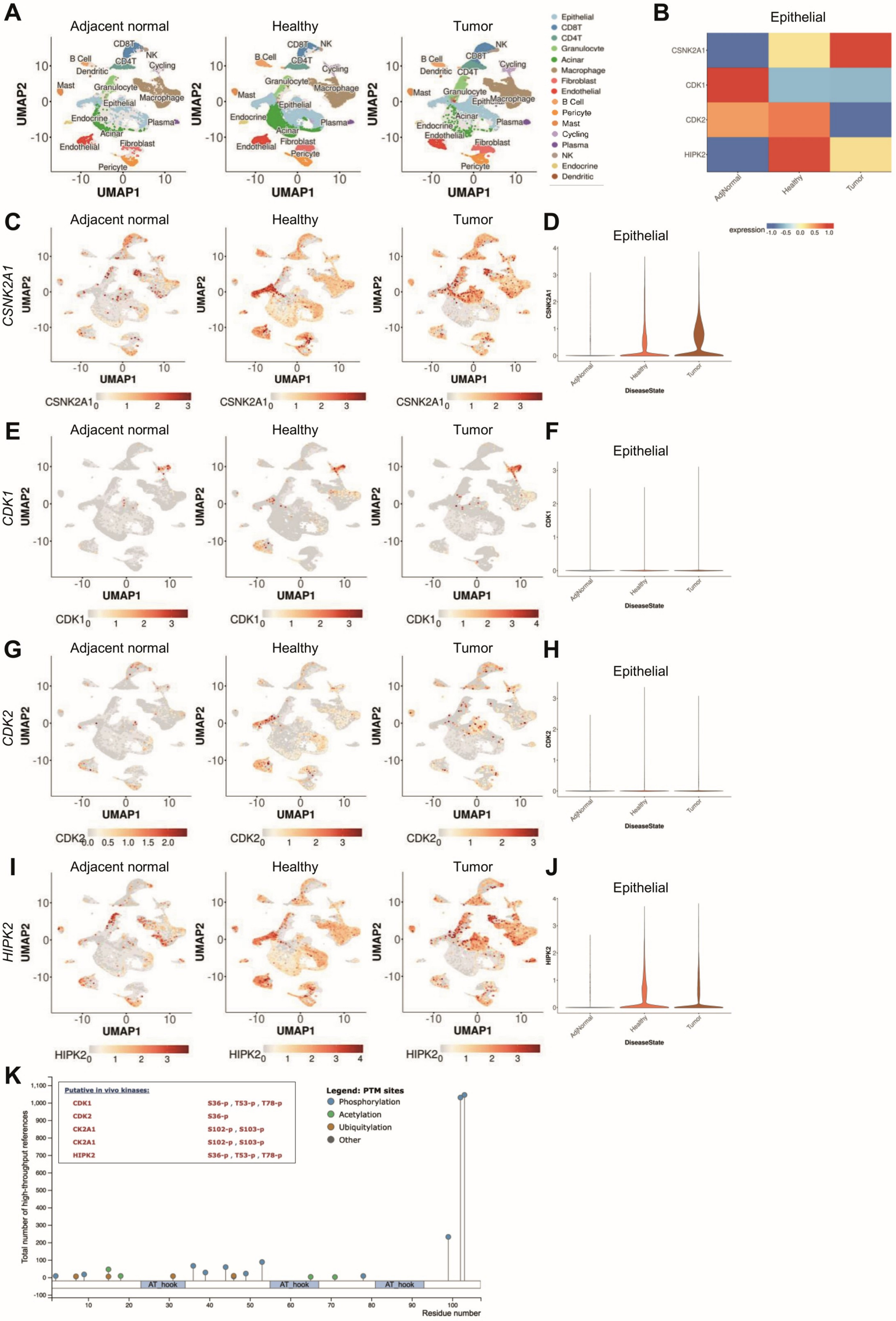
A
Adjacent normal
Healthy
Tumor
UMAP1
UMAP2
CD8T
NK
B Cell
CD4T
Cycling
Dendritic
Mast
Granulocyte
Macrophage
Epithelial
Endocrine
Plasma
Acinar
Endothelial
Fibroblast
Pericyte
Epithelial
CD8T
CD4T
Granulocyte
Acinar
Macrophage
Fibroblast
Endothelial
B Cell
Pericyte
Mast
Cycling
Plasma
NK
Endocrine
Dendritic
B
Epithelial
CSNK2A1
CDK1
CDK2
HIPK2
AdjNormal
Healthy
Tumor
expression
-1.0 -0.5 0.0 0.5 1.0
C
CSNK2A1
D
Epithelial
DiseaseState
E
CDK1
F
G
CDK2
H
I
HIPK2
J
K
Putative in vivo kinases:
CDK1 S36-p , T53-p , T78-p
CDK2 S36-p
CK2A1 S102-p , S103-p
CK2A1 S102-p , S103-p
HIPK2 S36-p , T53-p , T78-p
Legend: PTM sites
Phosphorylation
Acetylation
Ubiquitylation
Other
Total number of high-throughput references
AT_hook
Residue number

Supplementary Figure 3: Identification of CK2 as the leading kinase phosphorylating the most frequent HMGA1 phosphorylation sites.

(A) UMAP visualization of pancreatic microenvironment cell types by disease state: Adjacent Normal (*n* = 3), Healthy (*n* = 6), and Tumor (*n* = 16). Data source: Pancreatic Tissue Single Cell Atlas. **(B)** Heatmap showing the expression levels of *CSNK2A1* (encoding the CK2 protein), *CDK1*, *CDK2*, and *HIPK2* in epithelial cells derived from Healthy, Adjacent Normal, and Tumor samples. Color scale represents normalized expression values ranging from blue (-1) to yellow (0) to red (+1). **(C, E, G, I)** UMAP visualizations showing *CSNK2A1*, *CDK1*, *CDK2* and *HIPK2* expression, respectively, across major cell populations subset by disease state (Adjacent Normal, Healthy, Tumor). Data source: Pancreatic Tissue Single Cell Atlas. **(D, F, H, J)** Violin plots comparing *CSNK2A1, CDK1*, *CDK2* and *HIPK2* gene expression, respectively, in epithelial cells from Adjacent Normal (n = 892), Healthy (n = 14,380), and Tumor (n = 9,484) samples. Data source: Pancreatic Tissue Single Cell Atlas. **(K)** The plot summarizes the most frequently detected PTM sites on HMGA1 from PhosphoSitePlus® (v6.8.0), along with putative *in vivo* kinases. The y-axis shows the number of high-throughput references (proteomic mass spectrometry), and the x-axis indicates the HMGA1 residue position. Only sites with ≥5 references are shown. Colors: light blue (phosphorylation), green (acetylation), orange (ubiquitylation), gray (other).

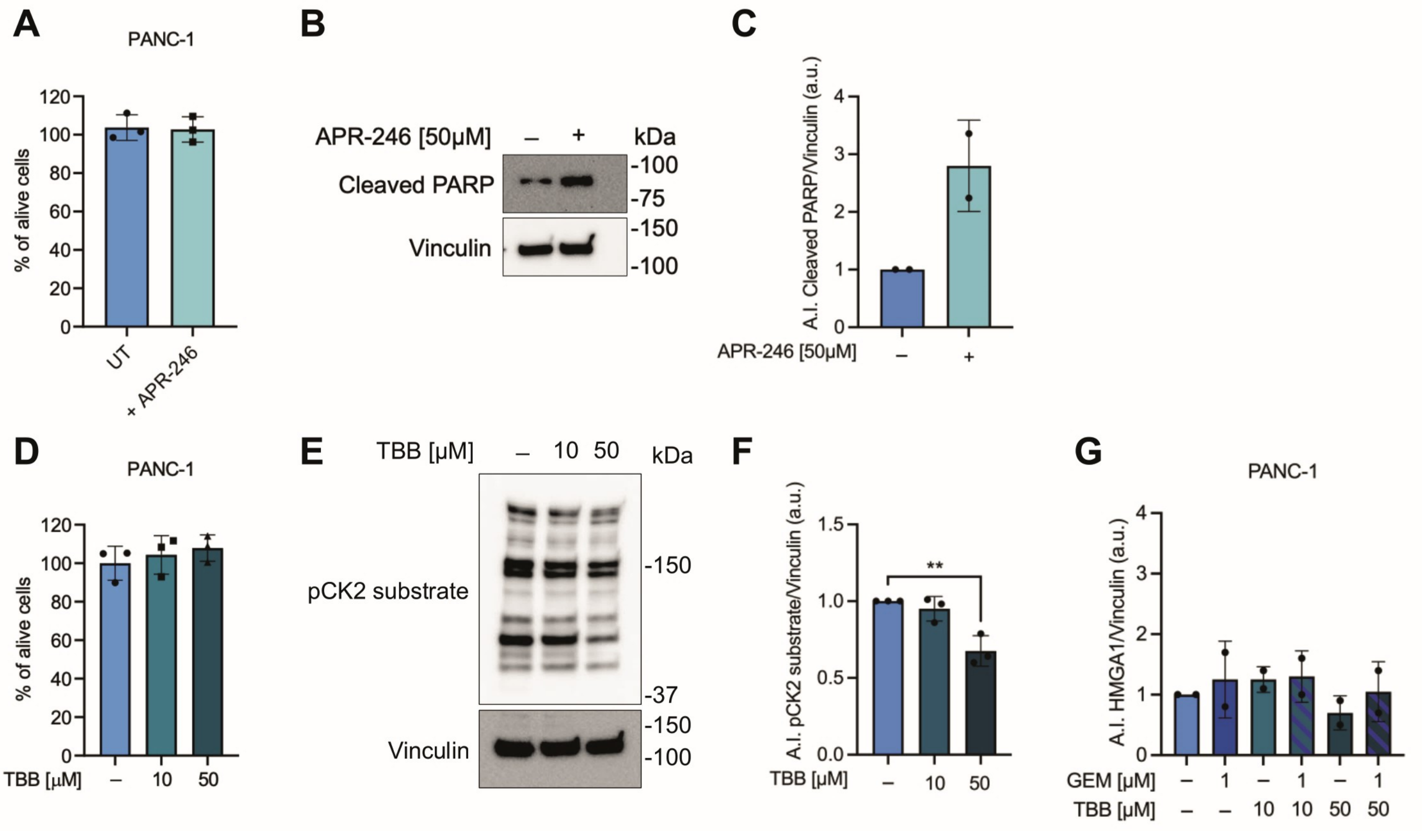

A
PANC-1
% of alive cells
120
100
80
60
40
20
0
UT
+ APR-246
B
APR-246 [50μM]
–
+
kDa
Cleaved PARP
-100
-75
Vinculin
-150
-100
C
A.I. Cleaved PARP/Vinculin (a.u.)
4
3
2
1
0
APR-246 [50μM]
–
+
D
PANC-1
% of alive cells
TBB [μM]
–
10
50
E
TBB [μM]
–
10
50
kDa
pCK2 substrate
-150
-37
Vinculin
-150
-100
F
A.I. pCK2 substrate/Vinculin (a.u.)
1.5
1.0
0.5
0.0
**
TBB [μM]
–
10
50
G
PANC-1
A.I. HMGA1/Vinculin (a.u.)
4
3
2
1
0
GEM [μM]
–
1
–
1
–
1
TBB [μM]
–
–
10
10
50
50

Supplementary Figure 4: Effect of APR-246 and TBB on cell viability and target protein expression in PANC-1 cells

(A) Bar chart of PANC-1 cells viability after 50 µM Eprenetapopt (APR-246) treatment. **(B)** Immunoblot of Cleaved PARP 48h after 50µM APR-246 treatment of PANC-1 cells. **(C)** Bar charts depict the average signal intensity (A.I.) in a.u. of Cleaved PARP versus Vinculin. Data plotted are mean of two independent experiments +/- SD. **(D)** Bar chart of PANC-1 cells viability after 10 or 50 µM 4,5,6,7-Tetrabromobenzotriazole (TBB) treatment. **(E)** Immunoblot of pCK2 substrate 24h after 10 or 50 µM TBB treatment of PANC-1 cells. **(F)** Bar charts depict the A.I. of pCK2 substrate versus Vinculin (a.u.). Data plotted are mean of three independent experiments +/- SD. (Unpaired t-test). ** $p<0.01$. **(G)** Bar charts depict A.I. (a.u.) of HMGA1 versus Vinculin analyzed using Image Lab Software (Bio-Rad, version 6.1.0 build 7). Data plotted are mean of two independent experiments +/- SD.

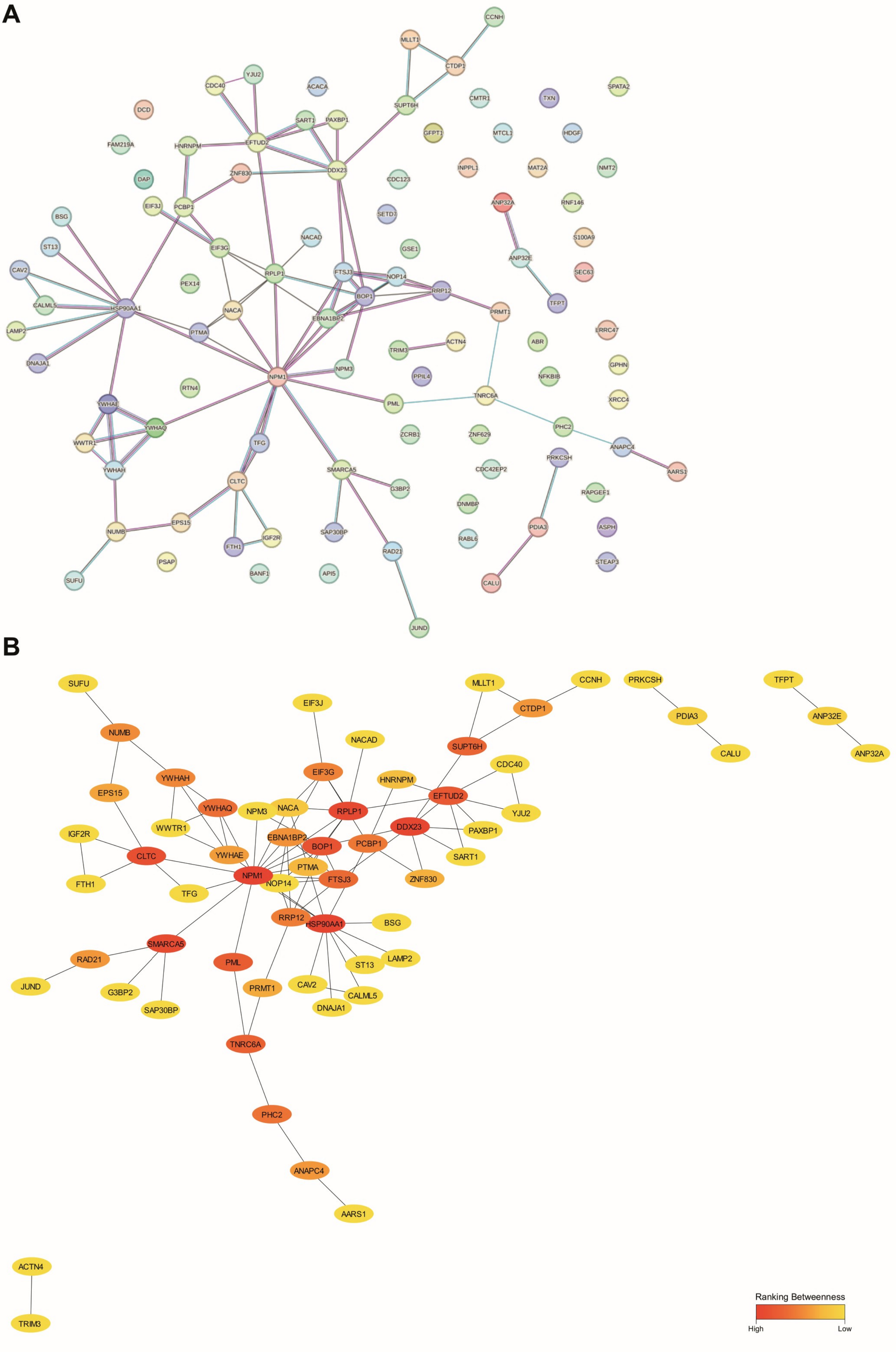
A
CCNH
MLLT1
CTDP1
YJU2
CDC40
ACACA
SUPT6H
CMTR1
TXN
SPATA2
DCD
SART1
PAXBP1
GFPT1
MTCL1
HDGF
FAM219A
HNRNPM
EFTUD2
ZNF830
DDX23
INPPL1
MAT2A
NMT2
DAP
CDC123
EIF3J
PCBP1
SETD7
ANP32A
RNF146
BSG
NACAD
S100A9
ST13
EIF3G
GSE1
ANP32E
CAV2
RPLP1
FTSJ3
NOP14
SEC63
PEX14
RRP12
BOP1
TFPT
CALML5
HSP90AA1
NACA
PRMT1
EBNA1BP2
LAMP2
PTMA
LRRC47
TRIM3
ACTN4
ABR
DNAJA1
NPM3
GPHN
PPIL4
NFKBIB
NPM1
RTN4
YWHAE
PML
TNRC6A
XRCC4
YWHAQ
ZCRB1
ZNF629
PHC2
WWTR1
TFG
ANAPC4
YWHAH
PRKCSH
SMARCA5
CDC42EP2
AARS1
CLTC
G3BP2
RAPGEF1
DNMBP
EPS15
NUMB
IGF2R
SAP30BP
PDIA3
ASPH
FTH1
RAD21
RABL6
PSAP
STEAP3
SUFU
BANF1
API5
CALU
JUND
B
SUFU
MLLT1
CCNH
PRKCSH
TFPT
CTDP1
EIF3J
PDIA3
ANP32E
NUMB
NACAD
SUPT6H
CALU
ANP32A
CDC40
EIF3G
HNRNPM
YWHAH
EFTUD2
EPS15
YWHAQ
NPM3
NACA
RPLP1
YJU2
DDX23
WWTR1
PAXBP1
IGF2R
EBNA1BP2
PCBP1
BOP1
YWHAE
SART1
CLTC
PTMA
ZNF830
NPM1
FTSJ3
FTH1
TFG
NOP14
RRP12
BSG
HSP90AA1
SMARCA5
LAMP2
ST13
RAD21
PML
JUND
CAV2
PRMT1
G3BP2
CALML5
SAP30BP
DNAJA1
TNRC6A
PHC2
ANAPC4
AARS1
ACTN4
TRIM3
Ranking Betweenness
High
Low

Supplementary Figure 5: Protein-Protein Interaction analysis of the significantly dysregulated proteins reveals NPM1 as a key hub protein in tumor cell proliferation. **(A)** STRING protein-protein interaction network of 101 significantly dysregulated proteins. The network includes 101 nodes and 93 edges. Circles represent query proteins, lines represent protein-protein associations. Line color indicates the type of interaction evidence: purple = experimentally determined, light blue = from curated databases, black = co-expression. Medium confidence (0.400) was used as confidence score. **(B)** Network of 63 hub genes from protein-protein interaction network. Genes were ranked by betweenness centrality mode. The node color reflects the degree of connectivity through a color scale ranging from red (high) to yellow (low).

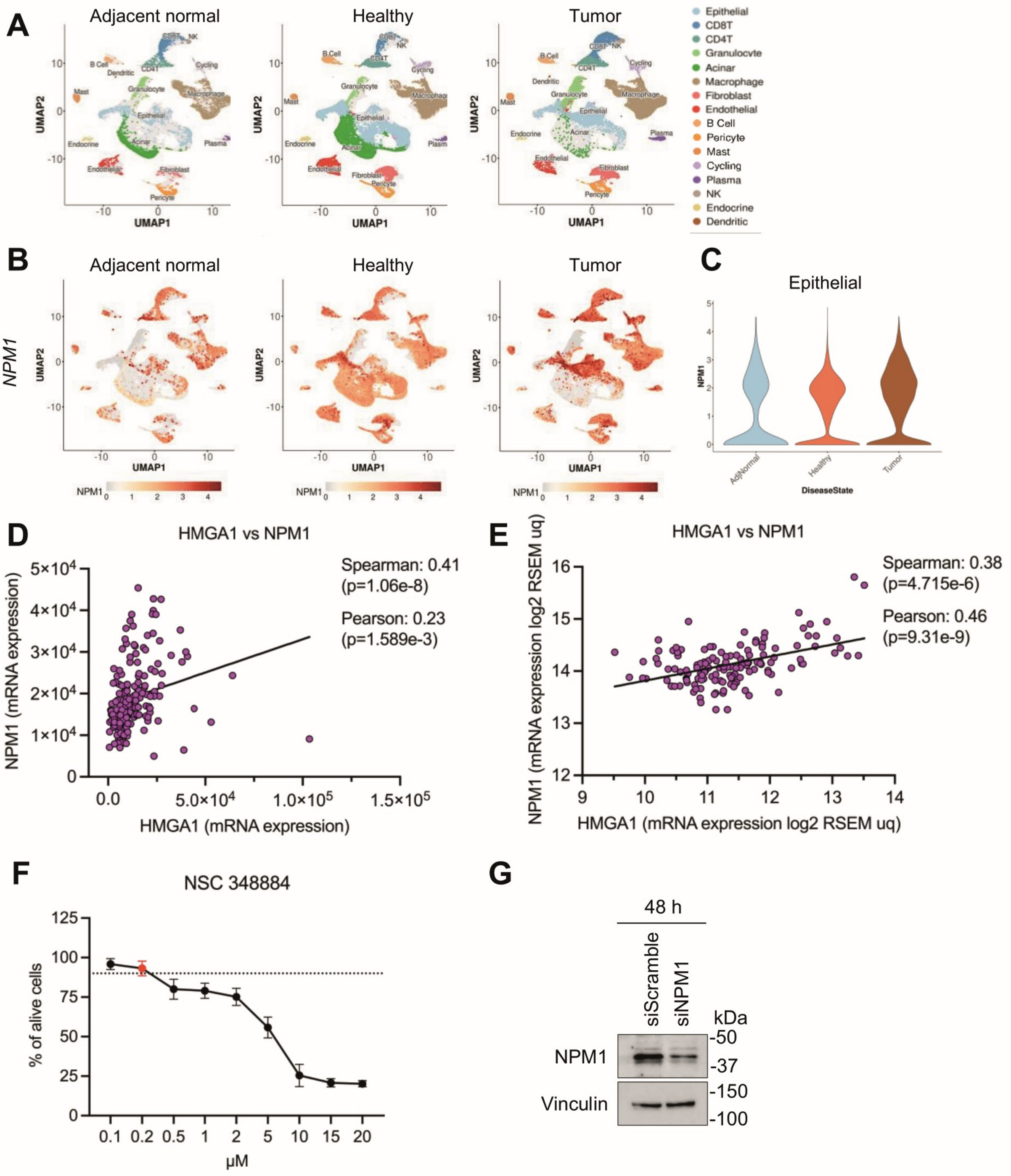
A
Adjacent normal
Healthy
Tumor
UMAP1
UMAP2
Epithelial
CD8T
CD4T
Granulocyte
Acinar
Macrophage
Fibroblast
Endothelial
B Cell
Pericyte
Mast
Cycling
Plasma
NK
Endocrine
Dendritic
B
NPM1
C
Epithelial
AdjNormal
Healthy
Tumor
DiseaseState
D
HMGA1 vs NPM1
Spearman: 0.41 (p=1.06e-8)
Pearson: 0.23 (p=1.589e-3)
NPM1 (mRNA expression)
HMGA1 (mRNA expression)
E
HMGA1 vs NPM1
Spearman: 0.38 (p=4.715e-6)
Pearson: 0.46 (p=9.31e-9)
NPM1 (mRNA expression log2 RSEM uq)
HMGA1 (mRNA expression log2 RSEM uq)
F
NSC 348884
% of alive cells
μM
G
48 h
siScramble
siNPM1
kDa
NPM1
Vinculin
-50
-37
-150
-100

Supplementary Figure 6: *NPM1* gene expression analysis in PDAC datasets.

(A) UMAP visualization of pancreatic microenvironment cell types by disease state: Adjacent Normal (n = 3), Healthy (n = 6), and Tumor (n = 16). Data source: Pancreatic Tissue Single Cell Atlas. **(B)** UMAP visualizations showing *NPM1* expression across the major cell populations subset by disease state (Adjacent Normal n = 3, Healthy n = 6, Tumor n = 16). Data source: Pancreatic Tissue Single Cell Atlas. **(C)** *NPM1* gene expression level in tumor derived epithelial cells compared to adjacent normal or healthy epithelial cells (nCells: 892 Adj.Normal; 14,380 Healthy; 9,484 Tumor). Data source: Pancreatic Tissue Single Cell Atlas. **(D)** Scatter plot showing the relationship between HMGA1 (x-axis) and NPM1 (y-axis) mRNA expression levels in pancreatic adenocarcinoma samples (TCGA, GDC dataset n = 179), accessed via cBioPortal. Each dot represents a single tumor sample. A regression line is shown, indicating a positive correlation (Spearman $\rho = 0.41$, $p = 1.06 \times 10^{-8}$; Pearson $r = 0.23$, $p = 1.59 \times 10^{-3}$). The observed co-expression supports a potential transcriptional association between HMGA1 and NPM1. **(E)** Scatter plot showing the relationship between HMGA1 (x-axis) and NPM1 (y-axis) mRNA expression levels (reported as log2 RSEM upper quartile normalized) in pancreatic ductal adenocarcinoma samples (CPTAC, Cell 2021 dataset n = 140), accessed via cBioPortal. Each dot represents a single tumor sample. A regression line is shown, indicating a positive correlation (Spearman $\rho = 0.38$, $p = 4.715 \times 10^{-6}$; Pearson $r = 0.46$, $p = 9.31 \times 10^{-3}$). **(F)** Identification of the highest dose of NSC348884 able to preserve the HMGA1 KO PANC-1 cells viability ≥ 90%. **(G)** Representative Western blot showing NPM1 protein levels 48 h after siRNA-mediated silencing. Vinculin was used as loading control.

Table legends

Supplementary Table 1. Mass spectrometry (MS) analysis of conditioned medium (CM) from PANC-1 cells following KD of mutant p53.

The table lists proteins identified by MS in the CM of PANC-1 pancreatic cancer cells after silencing of mutant p53.

Supplementary Table 2. Phosphoproteomic analysis of HMGA1 KO PANC-1 cells treated with conditioned medium (CM) from PANC-1 and HMGA1 KO PANC-1 cells.

The table lists phosphoproteins identified in HMGA1 knockout (KO) PANC-1 cells following treatment with CM derived from either PANC-1 or HMGA1 KO PANC-1 cells. Changes in phosphorylation were analyzed to assess the impact on intracellular phosphorylation events.
